# Irinotecan/scFv co-loaded liposomes coaction on tumor cells and CAFs for enhanced colorectal cancer therapy

**DOI:** 10.1186/s12951-021-01172-0

**Published:** 2021-12-14

**Authors:** Zhaohuan Li, Chunxi Liu, Chenglei Li, Fangqing Wang, Jianhao Liu, Zengjuan Zheng, Jingliang Wu, Bo Zhang

**Affiliations:** 1 School of Pharmacy, Weifang Medical University Weifang, Shandong, 261053 People’s Republic of China; 2 Department of Pharmacy, Qilu Hospital, Cheeloo College of Medicine, Shandong University, Ji’nan, 250012 Shandong People’s Republic of China; 3 School of Bioscience and Technology, Weifang Medical University, Weifang, 261053 Shandong People’s Republic of China

**Keywords:** Colorectal cancer, Liposomes, Cancer-associated fibroblasts, Single chain fragment variable, Co-cultured cells

## Abstract

**Background:**

Cancer-associated fibroblasts (CAFs), as an important component of stroma, not only supply the “soils” to promote tumor invasion and metastasis, but also form a physical barrier to hinder the penetration of therapeutic agents. Based on this, the combinational strategy that action on both tumor cells and CAFs simultaneously would be a promising approach for improving the antitumor effect.

**Results:**

In this study, the novel multifunctional liposomes (IRI-RGD/R9-sLip) were designed, which integrated the advantages including IRI and scFv co-loading, different targets, RGD mediated active targeting, R9 promoting cell efficient permeation and lysosomal escape. As expected, IRI-RGD/R9-sLip showed enhanced cytotoxicity in different cell models, effectively increased the accumulation in tumor sites*,* as well as exhibited deep permeation ability both in vitro and in vivo. Notably, IRI-RGD/R9-sLip not only exhibited superior in vivo anti-tumor effect in both CAFs-free and CAFs-abundant bearing mice models, but also presented excellent anti-metastasis efficiency in lung metastasis model.

**Conclusion:**

In a word, the novel combinational strategy by coaction on both “seeds” and “soils” of the tumor provides a new approach for cancer therapy, and the prepared liposomes could efficiently improve the antitumor effect with promising clinical application prospects.

**Graphical Abstract:**

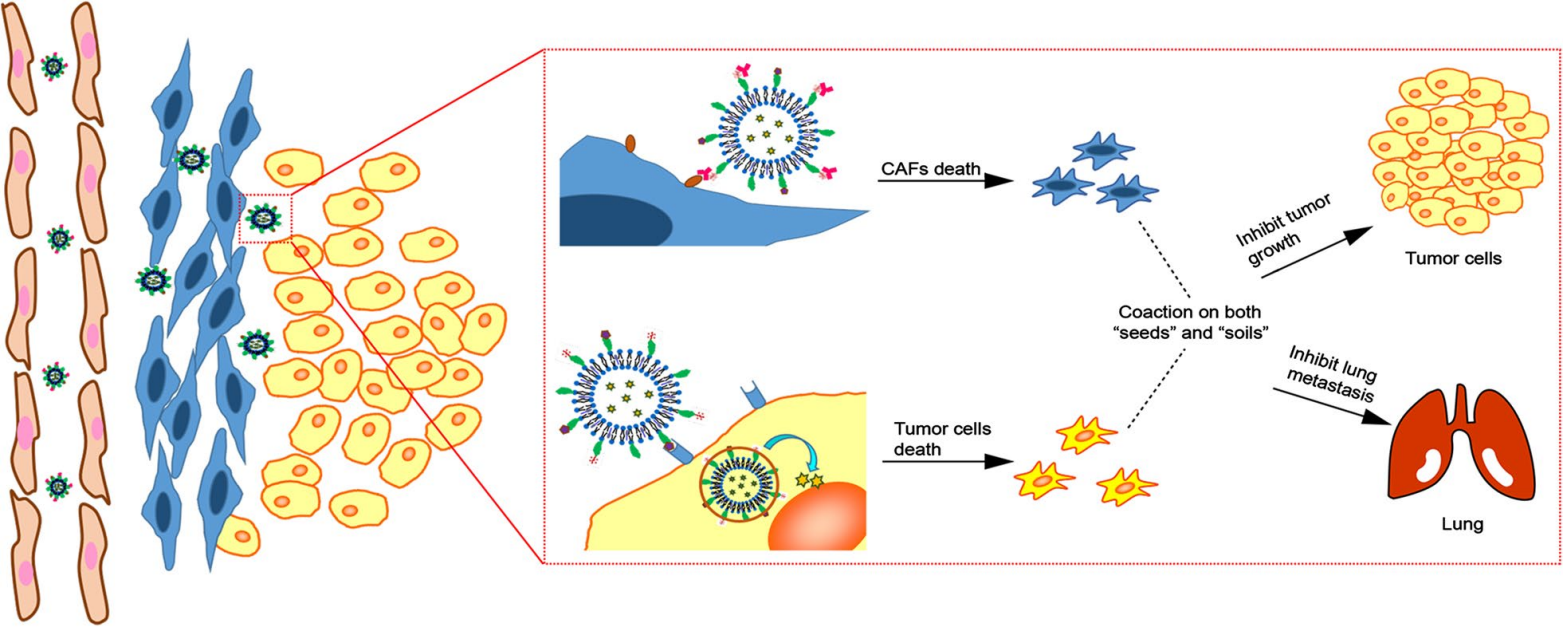

**Supplementary Information:**

The online version contains supplementary material available at 10.1186/s12951-021-01172-0.

## Background

Colorectal cancer (CRC) has become the third most common cancer with high morbidity and mortality [1, 2]. Chemotherapy is one of the important treatment manners in clinic, for example, Irinotecan (IRI), as the first-line chemotherapeutic agent for colorectal cancer, has been approved into market by many countries [3, 4]. IRI will be metabolized into pharmacological activated 7-ethyl-10-hydroxy camptothecin (SN-38), which could inhibit topoisomerase I activity, and result in double-stranded DNA rupture [5]. However, the application of chemotherapy is far from success, which does not significantly improve patients' cure rates or long-term survival [6]. With the deep understanding in cancer, it is well recognized that tumor microenvironment (TME) plays a crucial role in supporting the growth and invasion of tumor cells. Tumor cells can be considered as the "seeds" of cancer, while TME would provide the nutrients and favorable environment as the “soils” [7–10]. Therefore, on the basis of eliminating the “seeds” by chemotherapeutics, it is necessary to further destroy the "soils" for tumor cells survival to reshape the TME and thus effectively reduce tumor metastasis and recurrence.

TME is composed of extracellular matrix and numerous of stromal cells [11, 12]. Among that, cancer associated fibroblasts (CAFs) are the important components in tumor stroma especially in CRC, which is derived from normal fibroblasts and activated during the crosstalk with tumor cells [13, 14]. CAFs can promote tumor genesis, metastasis, and drug resistance by secreting the cytokines and extracellular matrix proteins such as α-smooth muscle actin (α-SMA) and fibroblast activator protein (FAP) [15–17]. FAP is a typeII transmembrane protein, which is highly expressed in CAFs of various cancers [18, 19]. In recent years, FAP has attracted widespread attention as an attractive target [20, 21]. Yu et al. designed the novel thermosensitive liposomes with FAP-α specific response, which not only killed tumor cells, but also destroyed stromal barrier to further promote deep tumor penetration [22]. Fan et al. developed one kind of pH-sensitive nanoparticles, which showed little cytotoxicity to normal tissue but could kill cancer cells and stromal cells efficiently [23]. In another study by Freedman et al. bispecific T-cell engagers (BiTE) were proposed, which could specifically kill the tumor cells as well as destroy the CAFs [24]. Single chain fragment variable (scFv) is a non-covalent heterodimer that specifically binds to FAP and inhibits the function of FAP [25, 26]. The use of FAP chimeric antibody scFv could eliminate the physical barrier formed by CAFs and inhibit cells metastasis. Herein, we attempt to explore whether the combination on IRI and scFv would be beneficial for enhanced CRC therapy by killing both tumor cells and CAFs simultaneously.

However, the combination therapy of IRI or scFv would face inevitable obstacles in many aspects. Firstly, it is hard for free drugs to produce synergistic effects due to the significant difference between small molecules and macromolecular drugs [27], accordingly, drug delivery system (DDS) is required to deliver both drugs to the tumor tissue simultaneously [28]. Secondly, after accumulated into the tumor, IRI and scFv would act on tumor cells and CAFs respectively, herein it is required to ensure each drug released and target to the acting site. Thirdly, due to the high interstitial fluid pressure in TME and the physical barrier formed by CAFs, the drug permeation into the deep tumor site will be severely impeded [29, 30]. Furthermore, after the drug endocytosed into the cells, it is easily degraded under the strong acidic environment of intracellular lysosomes, which makes IRI difficulty to internalize into the nucleus to exert therapeutic effects [31, 32]. Therefore, it is crucial to establish a novel DDS that could integrate the advantages in co-delivery of two drugs, responsible-release from different targets, high-efficient deep permeation, and lysosomal escape to overcome the above bottlenecks.

**Scheme 1:**
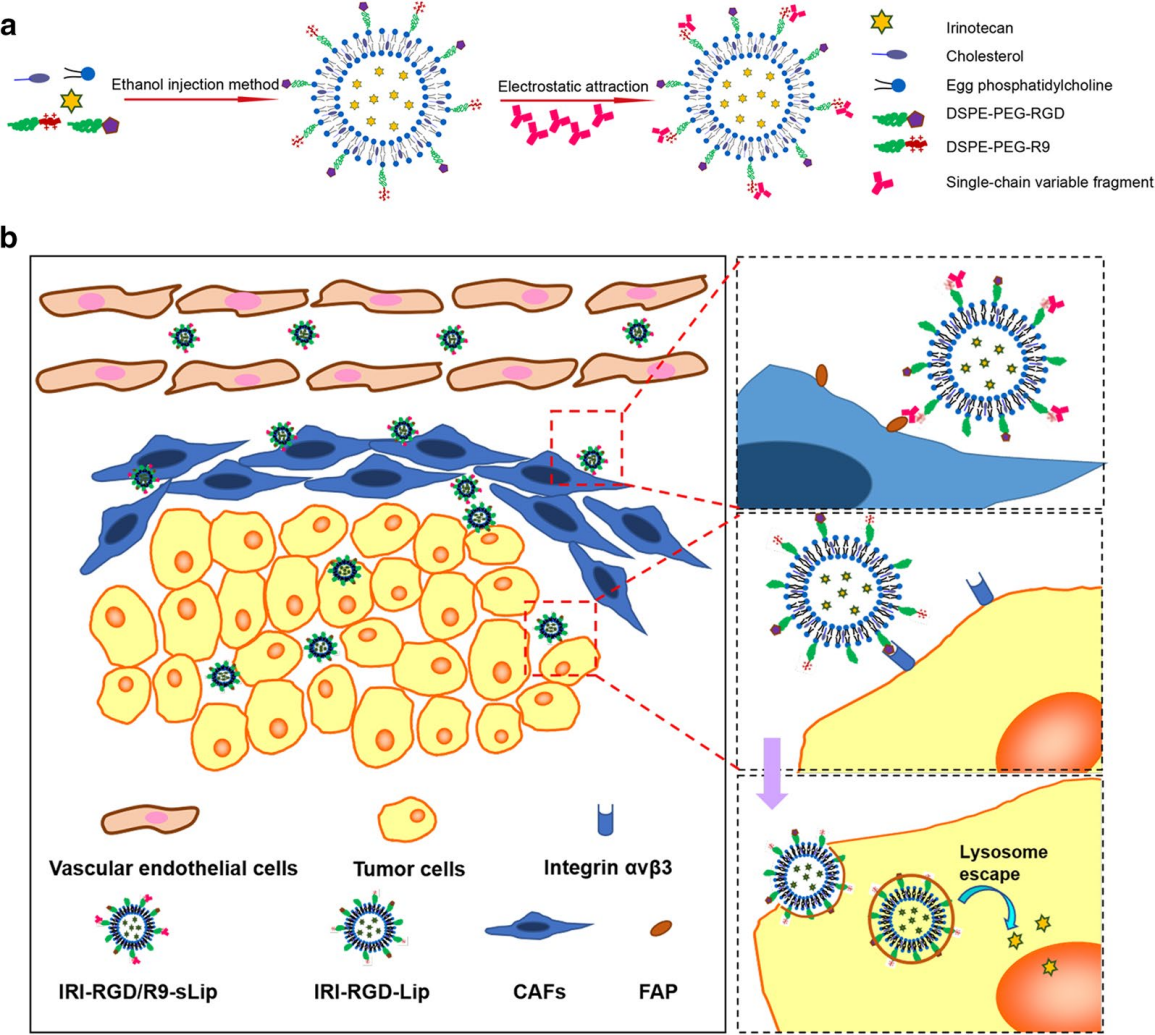
Design and combinational therapy process of multi-functional liposomes (IRI-RGD/R9-sLip). **a** The preparation process of liposomes. **b** In vivo process of IRI-RGD/R9-sLip

Liposomes have nowadays become one of the promising drug delivery systems with desired safety and clinical translational prospects [33–35]. In this study, we designed one kind of novel multi-functional liposomes (IRI-RGD/R9-sLip) for the co-delivery of chemotherapeutic drug IRI and anti-FAP scFv (Scheme 1). The liposomes were further modified with RGD ligand and 9-arginine cationic peptide (R9), in which RGD can specifically recognize integrin receptors on the surface of tumor cells, while R9 has been proven to have excellent functions in cells penetration and lysosomal escape [36–38]. As illustrated in Scheme 1, scFv was adsorbed on the surface of liposomes by electrostatic interaction with R9 and thus shielded the positive charge, which could keep stable during circulation. After accumulated into the tumor tissue, scFv could specifically target FAP to inhibit the proliferation of CAFs and eliminate the physical barrier of tumors. Importantly, due to the stronger binding force between FAP and its antibody (scFv), scFv was shed from the liposomes [39]. Subsequently, the uncovered cationic liposomes could further internalize into the tumor cells by RGD targeting and the excellent transmembrane ability of R9 can improve the deep penetration of liposomes [40, 41]. In addition, after the liposomes endocytosed into tumor cells, the amino group in R9 can further neutralize the acidic environment of the lysosomes through protonation and realize lysosomal escape of the liposomes, accordingly favoring IRI into the nucleus to inhibit the cell proliferation.

In the present work, multi-functional liposomes (IRI-RGD/R9-sLip) were prepared and evaluated for enhanced CRC therapy. Notably, in order to mimic the real CAFs-abundant TME, a novel co-cultured cells model was established by mixing NIH/3T3 cells with tumor cells to induce the activated CAFs. Particle sizes, zeta potentials and morphology of the liposomes were firstly characterized. Afterwards, the cellular uptake efficiency of liposomes was evaluated to verify cRGD targeting. The penetration capability of R9 was studied in vitro by establishing 3D microtumors and in vivo by frozen sections, while the lysosomal escape of R9 was confirmed by observing the distribution of liposomes in lysosomes. The in vivo antitumor effect of liposomes was assessed in two different tumor bearing mouse models, meanwhile, lung metastatic mouse model was established for anti-metastasis evaluation. In summary, the novel combinational strategy by coaction on both “seeds” and “soils” of the cancer was proposed in this study, which provides a new idea for cancer therapy. It is the first attempt to prepare multifunctional liposomes that integrated co-delivery, different targets, deep permeation and lysosomal escape into one system, which could efficiently improve the antitumor effect and hold promising clinical application prospects.

## Materials and methods

### Materials

1,2-distearoyl-sn-glycero-3-phosphoethanolamine-N [amin (polyethylene glycol)-2000] (DSPE-PEG_2000_-NH_2_) and Egg phosphatidylcholine (EPC) were obtained from AVT Pharmaceutical Technology Co., Ltd. (Shanghai, China). Lyso-Tracker Green was from Beyotime Biotechnology Co., Ltd. (Shanghai, China). Single-chain variable fragment (scFv) was provided by Merry Bio Co., Ltd. (Nanjing, China). Cholesterol, Hoechst33258, 3-(4,5-dimethylthiazol-2-yl)-2,5-diphenyltetrazolium bromide (MTT), Agarose and 4,6-diamino-2-phenylindole (DAPI) were acquired from Solarbio (Beijing, China). Nine arginine (R9) peptide was synthesized from Shanghai Taopu Biotechnology Co., Ltd. (Shanghai, China). DSPE-PEG-cRGD was synthesized by Xi’an Ruixi Biological Technology Co., Ltd. (Xi’an, China). Irinotecan base (IRI), Doxorubicin Hydrochloride (DOX·HCl) were purchased from Meilun Biotechnology Co., Ltd. (Dalian, China). FAP1 Ab-AF5344 and alpha-SMA Ab-AF1032 were obtained from Affinity Biosciences LTD (Jiangsu, China).

### Cell culture and animals

Embryonic mouse fibroblasts (NIH 3T3) and mouse CT-26 colon carcinoma cells were obtained from cell bank of Chinese Academy of Sciences (Shanghai, China). Both cells were cultured in the culture media containing 10% fetal bovine serum with addition of 1% penicillin–streptomycin. DMEM and RPMI-1640 media were selected for NIH 3T3 and CT-26 cells, respectively. The cells were cultured at 37 ºC containing 5% CO_2_. For the incubation of CT-26/NIH 3T3 co-cultured cells, both cells were mixed at the ratio of 1:2.

Female BALB/c mice (14–16 g) were provided by Pengyue Experimental Animal Co., Ltd. (Jinan, China). All animal experiments were conducted in accordance with the Regulations on Animal Control issued by the Ministry of Health of the People's Republic of China and the Ethical Review of Animal Experiments issued by Weifang Medical University (2017–025).

### Synthesis of DSPE-PEG-R9

R9 peptide were dissolved in DMSO and activated by EDC for 10–15 min, followed by addition of NHS with stirring for 2 h. Afterwards, DSPE-PEG2000-NH_2_ and a small amount of triethylamine were added to react for 24 h under the protection of nitrogen. The reaction solution was dialyzed for 48 h and then lyophilized to obtain DSPE-PEG-R9. The structure of DSPE-PEG-R9 was characterized by ^1^H-NMR. The yield and reaction percent were calculated as follows:

$$Yield \left( \% \right) = \frac{mass \,of \,DSPE - PEG - R9}{{mass\, of\, total}} \times 100\%$$

$${Reaction}\, {percent}\left( \% \right) = \frac{{{Area}({R}9)/{Number}({R}9)}}{{{Area}({PEG})/{Number}({PEG})}} \times 100\%$$

In which Area_(PEG)_ and Area_(R9)_ represent the peak area of “alkyl in PEG” and “guanidyl in R9”, repectively. While Number_(PEG)_ and Number_(R9)_ represent the hydrogen proton numbers of “alkyl in PEG” and “guanidyl in R9”, respectively.

### Preparation of liposomes

Before the preparation of liposomes, DSPE-PEG-R9 was obtained by one-step amide reaction using DSPE-PEG_2000_-NH_2_ and R9 peptide. IRI-RGD/R9-Lip were prepared via the following procedures. IRI, EPC, DSPE-PEG-cRGD, DSPE-PEG-R9 and cholesterol were dissolved in 2 mL ethanol to form a lipophilic solution, in which the mole ratio of DSPE-PEG-cRGD: DSPE-PEG-R9: EPC was controlled at 5:5:90. The solution were slowly injected into 5 mL PBS and stirred at 60 ºC for 1 h. IRI-loaded liposomes (IRI-RGD/R9-Lip) were prepared after ultrasonication and filtered through 0.45 μm and 0.22 μm membrane for 3 times, respectively [42]. Afterwards, scFv was added and electrostatic adsorbed on the surface of IRI-RGD/R9-Lip to obtain co-loaded liposomes (IRI-RGD/R9-sLip).

Besides, IRI-loaded liposomes without cRGD and R9 modification (IRI-Lip) were prepared as control group. IRI-RGD-Liposomes (IRI-RGD-Lip) were also prepared with the mole ratio of DSPE-PEG-cRGD: EPC at 5:95. Additionally, doxorubicin base (DOX) was obtained after DOX·HCl was dehydrochlorinated, and DOX loaded liposomes (DOX-Lip) were prepared for fluorescent trace. All the liposomes were prepared using the same method.

### Characterization of liposomes

The morphologies of IRI-RGD/R9-sLip was visualized by transmission electron microscopy (TEM). Particle sizes, polydispersity index (PDI) and zeta potentials of liposomes were determined by Malvern Zetasizer Nano ZS90. To evaluate the stability of IRI-RGD/R9-sLip, the size changes was analyzed for 14 days after the samples diluted with RPMI-1640 or PBS, respectively. Besides, after the liposomes demulsified by 10% Triton X-100, the concentrations of IRI or DOX were measured by ultraviolet (UV) spectrophotometer at the UV absorption wavelength of 360 nm or 480 nm, respectively. Encapsulation efficiency (EE%) and drug loading (DL%) were figured out using the equations as below:

$$EE\% = \frac{{W_{encapsulated drug} }}{{W_{total drug} }} \times 100\%$$

$$DL\% = \frac{{W_{encapsulated drug} }}{{W_{liposomes} }} \times 100\%$$

### In vitro release of IRI and scFv

The release profiles of IRI were studied using the dialysis bag method, in which 0.5% Tween-80 were added into PBS (pH 7.4) solution to meet the sink condition [43]. Briefly, 1 mL free IRI or IRI-Lip were transferred into dialysis bags (MWCO = 3500) respectively and incubated with 30 mL releasing medium at 37 ºC under 100 rpm shaking. At each time interval, 2 mL solution was extracted and supplemented with fresh release medium of the equal volume. The drug contents were analyzed by UV spectrophotometer at the absorption wavelength of 360 nm.

Regarding the release profiles of anti-FAP scFv, in consideration only after binding to the FAP on CAFs, scFv would be shed from the liposomes. Herein, 300μL R9-sLip were firstly incubated with activated NIH3T3 cells, and PBS group was set as control group to eliminate the influence of cell secretions. At different time points, 150μL cell supernatant solution was extracted and diluted with 150μL PBS. The content of scFv was determined by BCA microprotein quantitative kit.

### In vitro cytotoxicity assay

In vitro cytotoxicity of free IRI, free scFv, IRI-Lip, IRI-RGD-Lip, IRI-RGD/R9-Lip and IRI-RGD/R9-sLip were evaluated using MTT assay. 150 μL of CT-26 cells (5 $\times$ 10^3^ cells/well) or CT-26/NIH 3T3 co-cultured cells (the ratio of CT-26 to NIH 3T3 cells at 1:2) were seeded in 96-well plates and cultured overnight [44]. Different concentrations of IRI or scFv were added respectively and incubated for 48 h. Afterwards, 20 μL of MTT (5 mg/mL) was added to each well and incubated for another 4 h. Finally, MTT medium was replaced by 150 μL of DMSO. The absorbance was measured at 490 nm using a microplate reader (ELX800, Bio-Tek, USA). Besides, blank liposomes were incubated with CT-26 cells or CT-26/NIH 3T3 co-cultured cells for 48 h to evaluate their cell viability.

The normal NIH 3T3 cells can be activated by tumor cells or tumor cell supernatant to become activated NIH 3T3 cells (CAFs). In brief, NIH 3T3 cells alone were cultured for 8 h and then pre-treated with supernatant of CT-26 cells for 16 h. After that, the cells were treated with IRI, RGD/R9-Lip and RGD/R9-sLip for 48 h and evaluated by MTT assay in activated NIH3T3 cells (incubated with CT-26 supernatants).

### Cell migration assay

Firstly, CT-26 cells (4 $\times$ 10^5^ cells/well) or CT-26/NIH 3T3 co-cultured cells were inoculated in six-well plates. After the cells grown to 90–100%, three lines were scraped from each plate with the sterile tip of a spear. Each well was washed twice with PBS and treated with different preparations. For the dosage of free IRI, IRI-Lip, IRI-RGD-Lip and IRI-RGD/R9-sLip, the concentration of IRI in all the formulations were fixed at 15 μg/mL. Inverted fluorescence microscopy was used to obtain the images at 0 h and 24 h. The migration rate was evaluated by area detection methods, which was measured by ImageJ software and calculated as

$${Migration} {rate} = \frac{{{Width}_{{0{h}}} - {Width}_{{24{h}}} }}{{{Width}_{{0{h}}} }} \times 100\%$$

### Western blot analysis

After washing cells with PBS for three times and adding cell lysis buffer, the cells were scraped off with a cell scrape and centrifuged at 14,000 g for 5 min. The concentration of the supernatant was quantified by Bicinchoninic acid (BCA) kit. The separated proteins that run on SDS-PAGE gels (12%) transferred to polyvinylidene difluoride (PVDF) membrane, and the sealant containing 5% milk was used to block the membrane for 2 h. After the membrane washed with TBST for three times, the primary antibody was added and incubated overnight. The membrane was further washed to remove the unbound primary antibody and then incubated with secondary antibody at room temperature for 1 h. Ultra-sensitive ECL chemiluminescence solution was added to observe the protein bands.

### In vitro cellular uptake

DOX-loaded liposomes were prepared to analyze the intracellular accumulation of drugs in substitution for IRI. CT-26 cells (5 × 10^4^ cells/well) were seeded in 24-well plates containing round glass sheet. The DOX-Lip or DOX-RGD-Lip at the DOX concentration of 10 μg/mL were added for 1 h. To study the effect of RGD on cells uptake efficiency, the cells were firstly incubated with free RGD solution at the concentration of 1 mg/mL for 4 h, followed by incubation with DOX-RGD-Lip for 1 h. Each plate was washed three times with PBS. 4% tissue fixing fluid was applied to immobilize cells and then washed away. The nuclei were stained with DAPI and washed three times with PBS. Finally, the round glass was placed on the slide, and observed using a confocal laser scanning microscope (CLSM). Besides, the cells were collected and the fluorescence intensity was quantified by flow cytometry.

### Endosomal escape

To verify the endosomal escape ability of DSPE-PEG-R9, CT-26 cells (2 × 10^5^cells/well) were seeded on confocal dish. After incubation for 24 h, the cells were incubated with DOX-Lip or DOX-R9-Lip for 0.5 h, 1 h, 2 h and 4 h. After washed with PBS, the cells were incubated with Lysotracker Green (70 nM) and Hoechst 33,258 for 30 min. The cells were observed under CLSM. The colocalization ratio of lysosome and liposome (Mander’s coefficients) was calculated by ImageJ [45, 46].

### 3D Tumor spheroids

Co-cultured cells (ratio of CT-26 to NIH 3T3 cells was 1:2, among which CT-26 2 × 10^3^ cells/well) were seeded onto 96-well plates with 1% agarose. After seven days of culture, a 3D tumor model was generated. DOX-Lip, DOX-RGD-Lip and DOX-RGD/R9-Lip were incubated with tumor spheroids for 6 h. DOX fluorescence signals at different depths were scanned by CLSM.

### Tumor penetration in vivo

The tumor-bearing mice model was established by subcutaneous injection of 0.1 mL CT-26/NIH 3T3 co-cultured cells (ratio of CT-26 to NIH 3T3 cells was 1:2, among which CT-26 1 × 10^7^ cells/mL) at the right hind legs in mice. When the tumor grew to approximately 200 mm^3^, DOX-Lip, DOX-RGD-Lip and DOX-RGD/R9-Lip were injected intravenously at a DOX concentration of 3 mg/kg. After 12 h, the mice were sacrificed and the tumors were fixed with 4% paraformaldehyde. The different depth sections of tumor tissues were cryo-sectioned by freezing microtome at the thickness of 10 μm, then the nuclei were stained with DAPI and the sections were observed by CLSM.

In addition, a 3D fluorescence imaging system was applied to further verify the penetration of R9. In briefly, CT-26/NIH 3T3 co-cultured cells tumor-bearing mice models were administrated with IR-780-Lip, IR-780-RGD-Lip and IR-780-RGD/R9-Lip at the IR-780 concentration of 0.1 mg/mL. At 8, 12 and 24 h post administration, the fluorescence distribution at the tumor site was observed by 3D fluorescence imaging system.

### In vivo animal imaging

BALB/c mice were subcutaneously injected with CT-26 cells (1 × 10^7^ cells/mL) or CT-26/NIH 3T3 co-cultured cells. IR-780 was selected as the imaging tracker in vivo due to its strong absorption at around 780 nm, which was beneficial to monitor the distribution in the body in real time. After the tumors grew about 100 mm^3^, free IR-780, IR-780-Lip, IR-780-RGD-Lip and IR-780-RGD/R9-sLip were injected intravenously at the IR-780 concentration of 0.1 mg/mL. At different time intervals post administration, the fluorescence imaging was observed using the in vivo imaging system (IVIS). 24 h later, the mice were sacrificed to dissect the organs and tumors for further ex vivo imaging.

Moreover, orthotopic tumor model was first attempted to further evaluate the in vivo biodistribution. BABL/c mice were anesthetized with chloral hydrate (4%) and the abdomen was cut to expose cecum. CT-26 cells (1 × 10^6^ cells suspended in 25μL of PBS/Matrigel, 1:1 v/v) were injected into the colon wall to establish orthotopic tumor model. After 10 days, free IR-780, IR-780-Lip, IR-780-RGD-Lip and IR-780-RGD/R9-sLip were injected intravenously at the IR-780 concentration of 0.1 mg/mL. At different time intervals post administration, the fluorescence imaging was observed using the in vivo imaging system (IVIS). 24 h later, the mice were sacrificed to dissect the organs and tumors for further ex vivo imaging.

### In vivo antitumor effects

The antitumor effects were evaluated in both CT-26 cells (1 × 10^7^ cells/mL) and CT-26/NIH 3T3 co-cultured cells tumor-bearing mice models, respectively. After the tumor grew to approximately 150 mm^3^, the mice were randomly divided into six groups (n = 5) and intravenously administrated with saline, free IRI, IRI-Lip, IRI-RGD-Lip, IRI-RGD/R9-Lip or IRI-RGD/R9-sLip every 2 days for 14 days (IRI was 20 mg/kg, scFv 0.334 mg/kg). The body weights and the tumor size were recorded every 2 days. Tumor volumes (V) were calculated as V = (tumor length × tumor width^2^)/2.

After treatment, the major organs were dissected from the sacrificed mice and the tumor weights were measured. Organs and tumors were fixed with 4% paraformaldehyde. All tissues were sectioned and embedded in paraffin, for hematoxylin and eosin (H&E) staining. Apoptosis of tumor tissues was detected by Colorimetric TUNEL Apoptosis Assay Kit. Ki-67 immunohistochemistry was performed according to the instructions to identify cell proliferation. Dewaxing and antigenic repair were performed on the sections. The tissue was blocked with bovine serum albumin (BSA). Subsequently, the expression of FAP or α-SMA was detected according to the instructions of rabbit polymer assay system. The above tissues were observed under microscope. The percentage of positive FAP or α-SMA area to the total area was quantified using ImageJ software.

Orthotopic tumor model was also attempted for further evaluation of in vivo antitumor effects. The mice were randomly divided into three groups (n = 3): (1) saline, (2) free IRI, (3) IRI-RGD/R9-sLip. After 7 times of administration, the mice were sacrificed and their colon tissues were collected for inhibitory evaluation. Afterwards, H&E staining was also carried out to explore the proliferative status of tumor.

### Evaluation of lung metastasis

Colorectal cancer is closely associated with distant metastasis, especially to the liver and lungs. To build a lung metastasis model of colorectal cancer, intravenous injection of CT-26 cells is widely applied in many studies [47–51]. Lung metastasis model was built by intravenously injecting of 0.1 mL CT-26 cells (1 × 10^7^ cells/mL) into the mice. All mice were randomly divided into 7 groups (n = 3) and the lungs of healthy mice served as negative control [47, 52]. One day later, mice with lung metastases were treated with Saline, free IRI, IRI-Lip, IRI-RGD-Lip, IRI-RGD/R9-Lip or IRI-RGD/R9-sLip (IRI 20 mg/kg, scFv 0.334 mg/kg). Changes in body weight of mice were detected during the treatment. After 7 times of administration, the mice were sacrificed and their lungs were collected for H&E staining and the numbers of lung nodules were recorded.

### Statistical analysis

All the data were presented as the mean ± standard deviation (SD). The differences between two groups were evaluated by Student’s t test using GraphPad Prism 8.0. *P* < 0.05 was considered to be statistical significance.

## Results and discussion

### Characterizations of liposomes

The structures of DSPE-PEG-cRGD and DSPE-PEG-R9 were verified by ^1^H-NMR. The chemical shift at 6.5–8.5 ppm was assigned to characteristic peaks of cRGD, indicating that DSPE-PEG-cRGD was successfully obtained (Additional file 1: Fig. S1a). Besides, the characteristic peaks at 7.0–8.0 ppm was assigned to guanidine group of R9, indicating that R9 was successfully conjugated to DSPE-PEG (Additional file 1: Fig. S1b). The yield of DSPE-PEG-R9 was 42% and the reaction percent of “R9 conjugated to DSPE-PEG-NH_2_” was 54%.

**Fig. 1:**
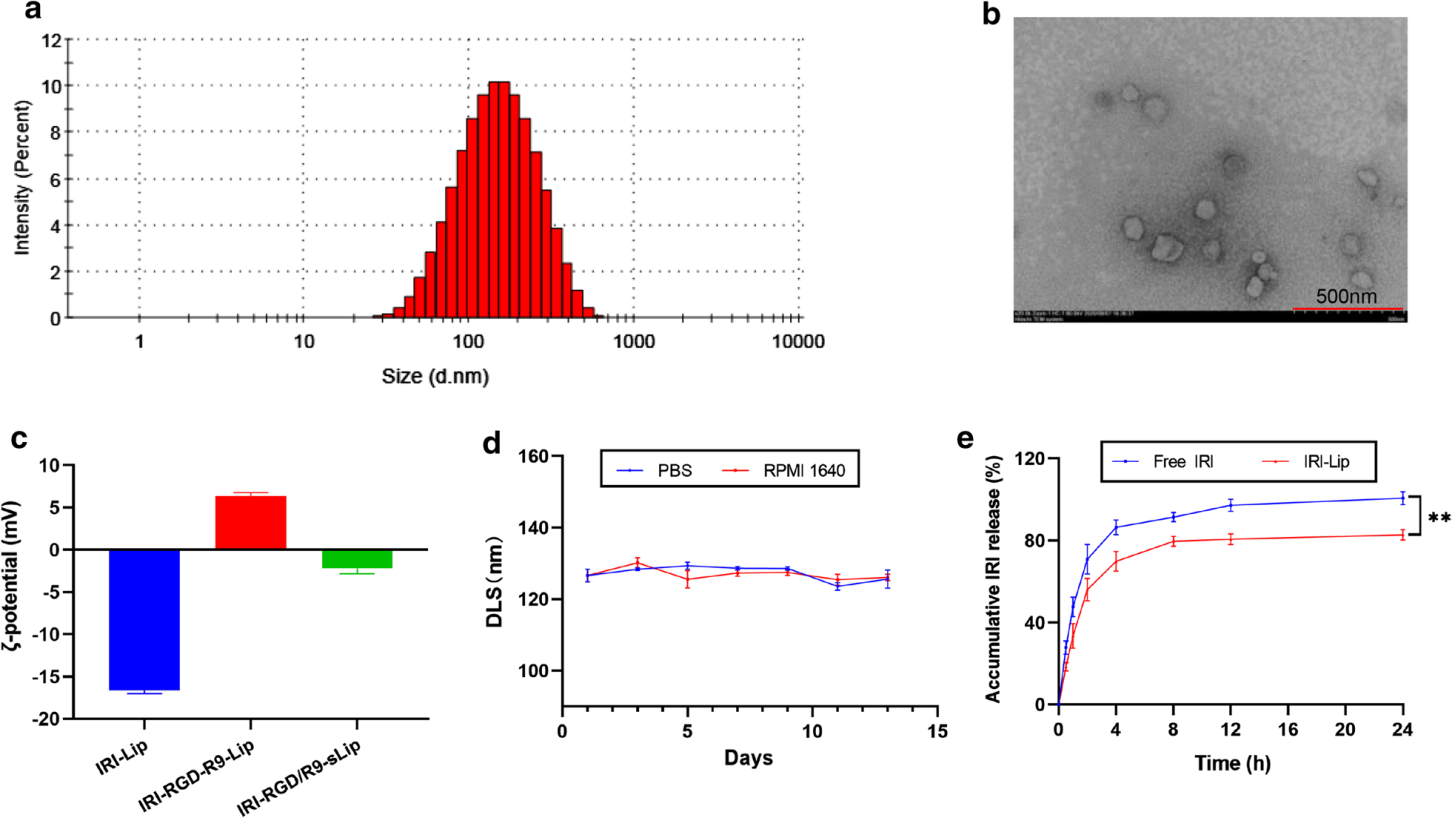
Characterization of liposomes. **a** Representative particle size distribution and **b** TEM image of IRI-RGD/R9-sLip. **c** Zeta-potential changes in various formulations. **d** The particle size changes of IRI-RGD/R9-sLip in PBS or RPMI 1640 medium within 14 days. **e** In vitro release profiles of Free IRI or IRI-Lip. ^**^P < 0.01

The preparation procedure of the liposomes was illustrated in Scheme 1A. Firstly, RGD and R9-modified cationic liposomes were prepared by ethanol injection, and then scFv was adsorbed on the surface of the cationic liposomes by electrostatic adsorption to form the co-delivery system. The zeta potential reversal of the liposomes further proved the successful synthesis of cationic liposomes and the successful adsorption of scFv on their surface (Fig. 1c).

The average particle size, PDI, zeta potentials, EE% and LE% of different liposomes were shown In Table 1. The mean particle sizes were less than 150 nm with PDI < 0.2, indicating the uniform dispersion of the liposomes. The average particle sizes and PDI of DOX-loaded and IR780-Liposomes were shown in Additional file 1: Table S1. The mean particle sizes were similar to IRI-loaded Liposomes. The zeta potential of IRI-RGD/R9-sLip was negative, which was beneficial for the liposomes to remain stable in circulation. IRI base could be readily encapsulated into the liposomes due to the hydrophobicity, with the encapsulation efficacies more than 90% in all IRI-loaded liposomes. TEM images showed regular sphericity (Fig. 1a, b). As shown in Fig. 1d, the stability of liposomes was tested in PBS or RPMI1640, and the size change was negligible within 14 days, suggesting that liposomes had good stability and fit for intravenous injection. The release behaviors of free IRI and IRI-Lip in PBS was shown in Fig. 1e. The release of free IRI showed obvious burst release, and its release exceeded 80% over 4 h. However, the release of IRI from IRI-Lip was significantly slower, which was conducive to the sustained release of drugs (*P* < 0.01). For scFv, it could be rapidly released from R9-sLip with the release amount nearly reached 100% in 2 h (Additional file 1: Fig. S6), indicating that the bind of scFv to FAP can trigger the scFv shedding off from R9-sLip.

**Table 1:** Characterizations of the liposomes

	DLS (nm)	PDI	Zeta potential (mV)	EE (%)	LE (%)
IRI-Lip	138.3 ± 2.0	0.13 ± 0.03	− 16.7 ± 0.3	90.4 ± 2.0	8.7 ± 1.3
IRI-RGD-Lip	141.7 ± 0.6	0.11 ± 0.02	− 16.5 ± 2.0	97.4 ± 3.3	9.9 ± 0.3
IRI-RGD/R9-Lip	125.1 ± 2.8	0.16 ± 0.02	+ 6.3 ± 0.3	100.2 ± 5.9	8.9 ± 0.5
IRI-RGD/R9-sLip	126.8 ± 2.6	0.19 ± 0.02	− 3.3 ± 0.4	98.0 ± 2.0	8.7 ± 0.1

### In vitro cytotoxicity

Cell viability of blank liposomes (RGD-Lip or RGD/R9-Lip) were first evaluated in both CT-26 cells and CT-26/NIH3T3 co-cultured cells, respectively. There was no significant cytotoxicity after RGD-Lip or RGD/R9-Lip incubation, in which the cell viabilities were above 85% (Additional file 1: Fig. S2a, b). These results suggested that the blank liposomes had good safety profiles. Besides, the cytotoxicity of RGD/R9-sLip, RGD/R9-Lip and free IRI were also evaluated by MTT assay in activated NIH3T3 cells (incubated with CT-26 supernatants). RGD/R9-Lip showed no significant toxicity, while RGD/R9-sLip had enhanced cytotoxicity than RGD/R9-Lip (Additional file 1: Fig. S3). These results indicated that scFv could inhibit the proliferation of CAFs. The cytotoxicity of irinotecan (IRI) in activated NIH3T3 cells was also evaluated. Additionally, the cells showed concentration dependent survival-rate after IRI treatment, indicating that IRI could also kill CAFs (Additional file 1: Fig. S4). It is due to that IRI is a nonselective cytotoxic drug that can kill any cells. From this aspect, the combination of IRI and scFv would be beneficial to improve the cytotoxicity on CAFs.

**Fig. 2:**
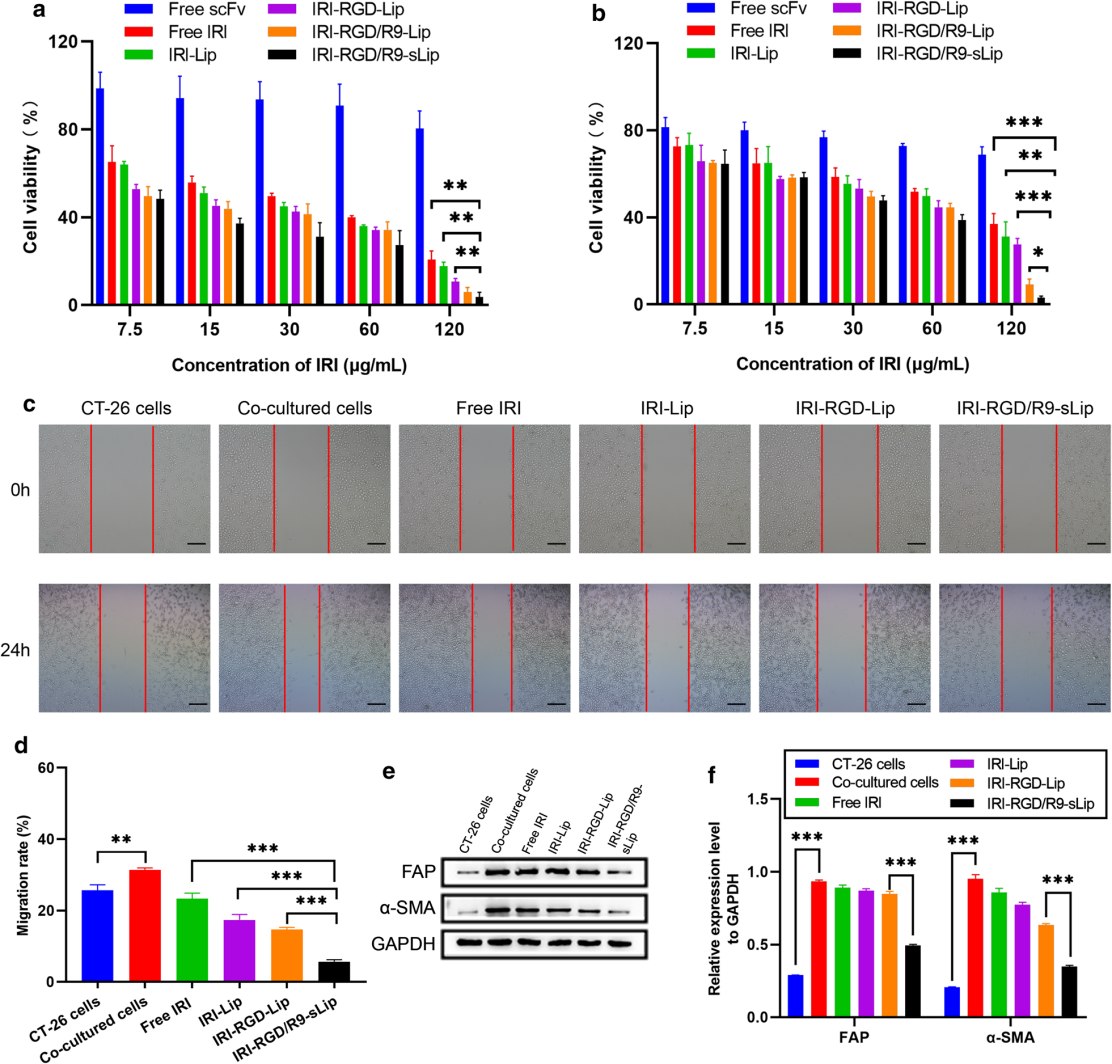
Analysis of cell proliferation and migration. Viability of **a** CT-26 cells and **b** co-cultured cells (ratio of CT-26 to NIH 3T3 cells was 1:2) after incubation with different formulations at different concentrations for 48 h. **c** Migration experiment after different formulations treatment for 24 h. Scale bar, 100 μm. **d** Cell migration percentage. **e** Western blot analysis of FAP and α-SMA expression levels and **f** quantification data. ^**^P < 0.01, ^***^P < 0.001

The cytotoxic effects of different drug-loaded formulations were evaluated after incubation with CT-26 cells or co-cultured cells for 48 h. In CT-26 cells, the survival rate in all groups showed concentration dependent profiles (Fig. 2a). IRI-RGD/R9-Lip showed obvious cytotoxicity at high concentration, which may be attributed to the favorable permeation and cellular uptake of R9. While on the other hand, no significant difference was observed in both IRI-RGD/R9-Lip and IRI-RGD/R9-sLip, indicating that scFv had little effect in CAFs-free cell model. In co-cultured cells, all of preparations exhibited varying degrees reduced inhibitory effect compared to that in CT-26 cells. Particularly, compared with IRI-RGD/R9-Lip, IRI-RGD/R9-sLip showed less cell viability with significant difference (Fig. 2b), which revealed that the addition of scFv could killing the CAFs and thus improved the cytotoxicity. In summary, these results suggested that CT-26 cells were more sensitive to chemotherapeutic agent, while the co-cultured cells showed increased tolerance to single chemotherapy. On the other hand, combination of IRI and scFv may reduce cell drug resistance by killing CAFs, and thus exhibited strong cytotoxicity.

### Wound healing study

CT-26 cells were set as the control group to evaluate the migration ability of cells in comparison to the co-cultured system. As shown in the Fig. 2c, d, the cell migration rate in CT-26/NIH 3T3 co-cultured cells was obviously increased than that in CT-26 cells, demonstrating that CAFs promoted cell migration. After treated with different formulations, cell migration ability showed different degrees of inhibition. Among that, the combination of IRI and scFv significantly inhibited cells migration and showed strongest anti-metastasis effect, which indicated that scFv could reduce cell migration rate by inhibiting CAFs.

FAP and α-SMA are the important biomarkers of CAFs with specifically up-regulated expression. Herein, the expression levels of FAP and α-SMA were detected by western blotting to verify the activation of CAFs in co-cultured cells. As shown in Fig. 2e, f, both FAP and α-SMA had weak expression in CT-26 cells, while obviously increased in co-cultured cells, suggesting that NIH 3T3 co-incubated with CT-26 cells could activate CAFs. Furthermore, after treatment by IRI, the expression of FAP and α-SMA decreased in different degrees. Compared with other formulations, the expression of FAP and α-SMA was obviously decreased after treatment by IRI-RGD/R9-sLip. These results indicated that the combination therapy could effectively inhibit the functions of CAFs, which was beneficial for inhibition of tumor metastasis.

### In vitro cellular uptake

**Fig. 3:**
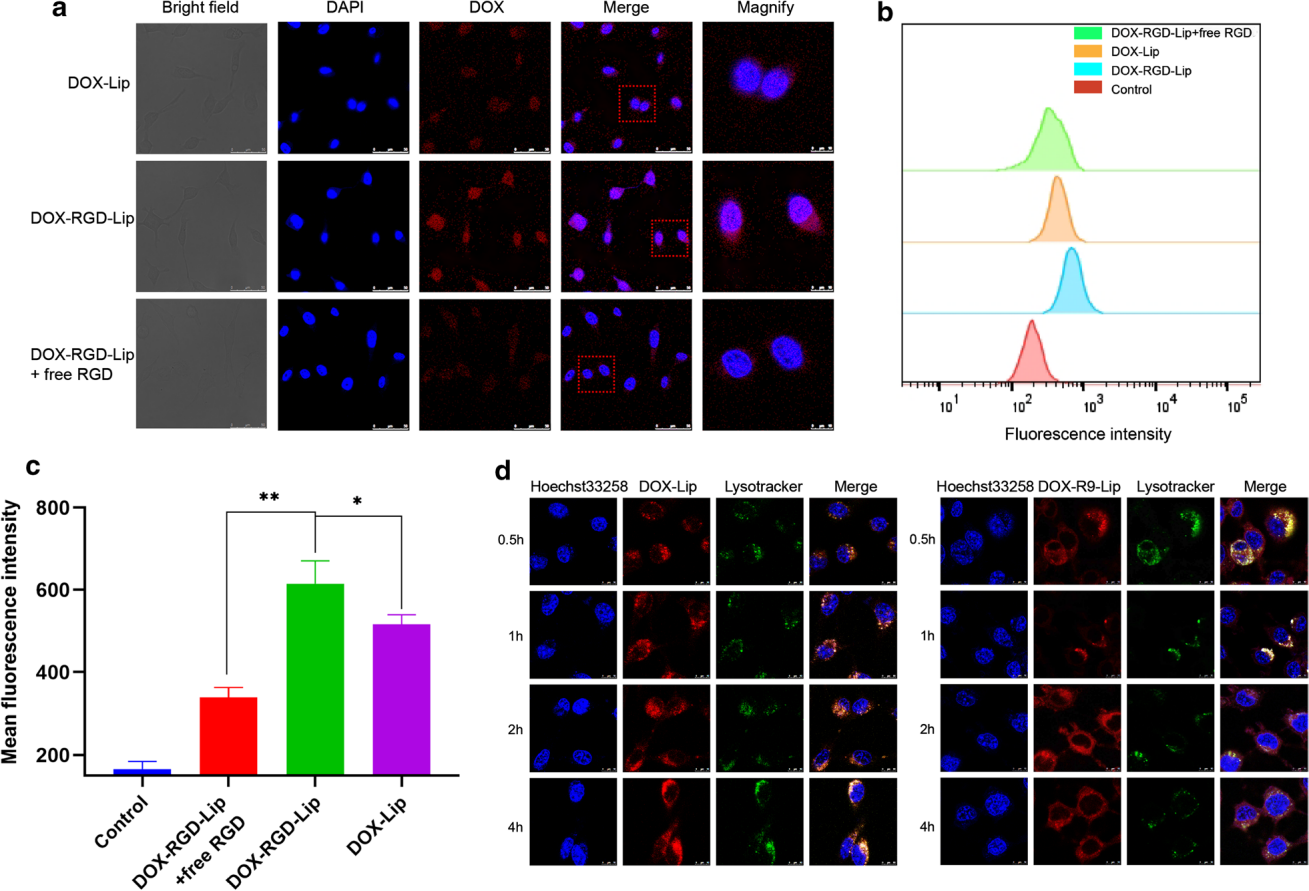
In vitro targeting and lysosomal escape assay. **a** CLSM qualitative analysis and **b** flow cytometry offset of CT-26 cells treated with different liposomes. **c** DOX fluorescence intensity was quantified by flow cytometric. Scale bar, 50 μm. ^*^P < 0.05, ^**^P < 0.01. **d** The distribution of DOX loaded liposomes in CT-26 cells. DOX-Lip and DOX-R9-Lip were incubated with CT-26 cells for 0.5 h, 1 h, 2 h and 4 h. Green color: lysosomes. Blue color: cell nuclei. Red color: DOX. Scale bar, 10 μm

CLSM and flow cytometry were performed to assess RGD mediated cellular uptake efficiency. Bule and red fluorescence represented nucleus and DOX, respectively. As shown in Fig. 3a, DOX-RGD-Lip group showed stronger red fluorescence than the DOX-Lip treatment group, indicating that RGD binds to integrin receptors of cells surface, thereby improving the targeting of liposomes. Furthermore, competitive inhibition test was performed to verify the enhanced uptake ability of RGD. The red fluorescence intensity decreased after excessive free RGD treatment in advance. This might be due to the saturation of cells surface receptors by free RGD, leading to reduced uptake efficiency of DOX-RGD-Lip. Similarly, the quantitative results in flow cytometry also proved that the fluorescence intensity of DOX in the DOX-RGD-Lip group was 1.2-fold higher than that of the DOX-Lip group and 1.8-fold higher than that of the DOX-RGD-Lip group treated with excessive free RGD in advance (Fig. 3b, c). The above results indicated that RGD-mediated endocytosis could effectively improve cell internalization.

### Endosomal escape

The lysosomal escape function of R9 peptide were verified by co-incubating DOX-R9-Lip with cells for different hours. Yellow spots represented the co-localization of DOX red fluorescence and lysosomal green fluorescence. After incubation for 0.5 h and 1 h, yellow spots with high fluorescence intensity were observed in the cytoplasm, indicating that liposomes were endocytosed into the lysosomes. While at 2 h, red fluorescence and green fluorescence were partially separated. At 4 h, only a small amount of red fluorescence overlapped with green fluorescence, indicating that liposomes were successfully escaped from lysosomes. In contrast, in the control group (DOX-Lip), even at 4 h, strong overlapped yellow fluorescence was observed (Fig. 3d). These results demonstrated that R9 modifies liposomes could help the drugs escape from lysosomes. Moreover, the colocalization ratio of lysosome and liposome (Mander’s coefficients) was calculated and illustrated in Additional file 1: Fig. S5, the percent of DOX-R9-Lip in lysosomes decreased with time increasing, meanwhile, the percent of DOX-R9-Lip in lysosomes is much lower than DOX-Lip in lysosomes (p < 0.01). All these results suggested that R9-modified liposomes would be beneficial for lysosome escape.

### In vitro and in vivo tumor penetration

**Fig. 4:**
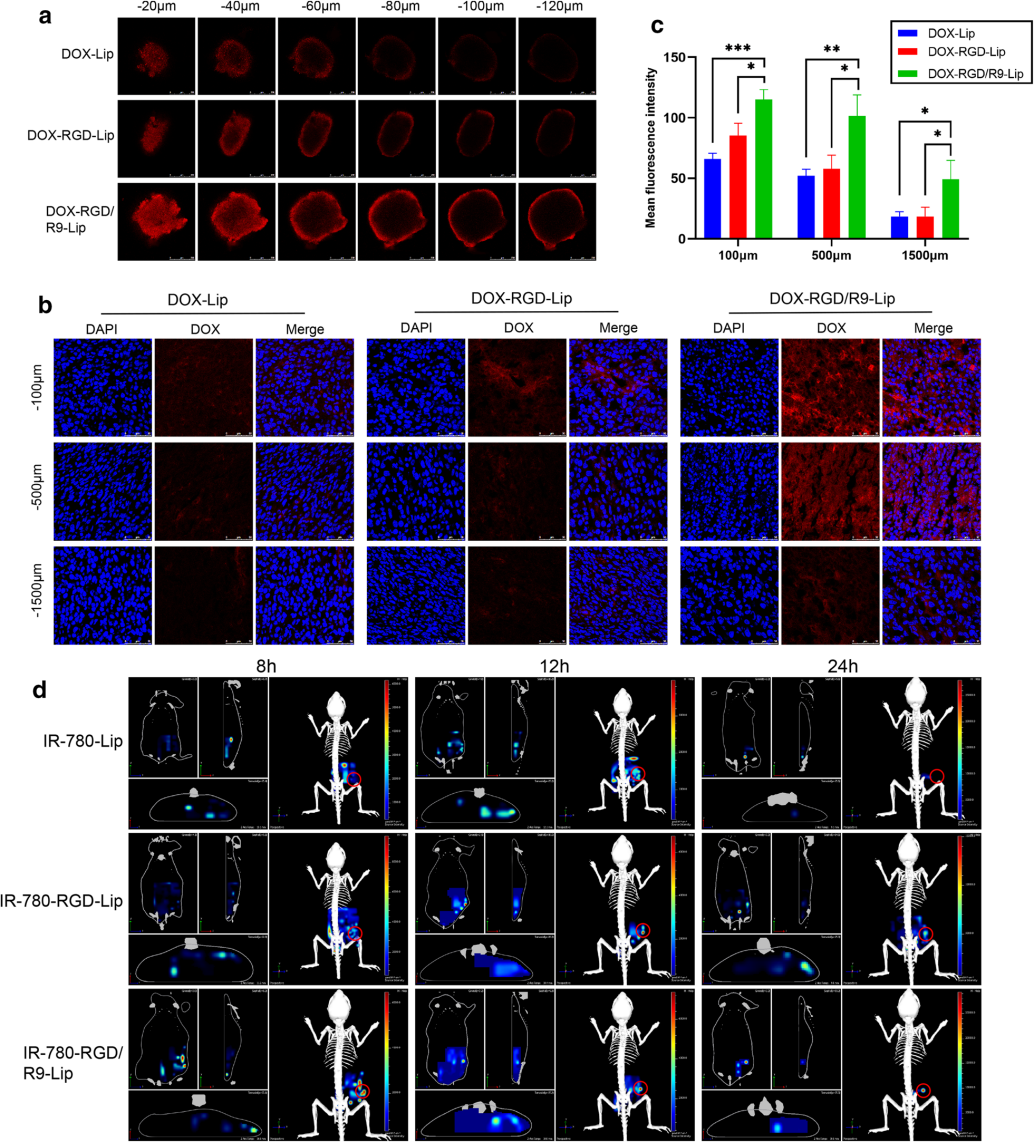
Permeability of DOX-RGD/R9-Lip in vitro and in vivo*.* **a** CLSM images of DOX-Lip, DOX-RGD-Lip and DOX-RGD/R9-Lip in vitro. **b** In vivo penetration of DOX-Lip, DOX-RGD-Lip and DOX-RGD/R9-Lip and **c** fluorescence intensity was quantified using Image J. **d** 3D image of IR-780-Lip, IR-780-RGD-Lip and IR-780-RGD/R9-Lip in vivo. ^*^P < 0.05, ^**^P < 0.01, ^***^P < 0.001. Scale bar, 250 μm

A novel in vitro 3D tumor sphere model constructed by co-cultured CT-26/NIH 3T3 cells were established to verify the penetration ability of R9-modified liposomes in vitro. DOX-Lip was used as a negative control and the red fluorescence represented DOX. As shown in Fig. 4a, compared with DOX-Lip group, DOX-RGD-Lip group showed enhanced permeability due to RGD targeting ability, while the fluorescence intensity increases of DOX-RGD/R9-Lip group at each depth than DOX-RGD-Lip, which was attributed to the R9 strong membrane penetration ability.

To further investigate the permeability of DOX-RGD/R9-Lip in vivo, subcutaneous tumor-bearing mice were administrated with DOX-Lip, DOX-RGD-Lip and DOX-RGD/R9-Lip by tail vein injection. As shown in Fig. 4b, DOX-Lip group and DOX-RGD-Lip group were distributed on the tumor surface, while the DOX-RGD/R9-Lip group could penetrate into deeper areas, and strong red fluorescence could be seen even at 1500 μm depth. In addition, the fluorescence intensity was quantified, in which DOX-RGD/R9-Lip at each depth was much higher than DOX-Lip and DOX-RGD/R9-Lip with significant differences (Fig. 4c). These results suggested that R9 could improve the penetration ability of liposomes and thus deliver the drug into deep tumor, which would be beneficial for efficiently killing the tumor cells.

In addition, the in vivo permeability of R9 was also further verified using a 3D imaging system. Weak fluorescence in IR-780-Lip indicated the poor penetration ability of normal liposomes, while enhanced permeability observed in IR-780-RGD-Lip group due to RGD active-targeting ability. Notably, IR-780-RGD/R9-Lip showed strong fluorescence at all the time points, suggesting that R9 could improve the penetration ability of liposomes (Fig. 4d). To exhibit the in vivo 3D imaging more visualized, a representive video file was attached in the Additional file 2.

### In vivo biodistribution study

**Fig. 5:**
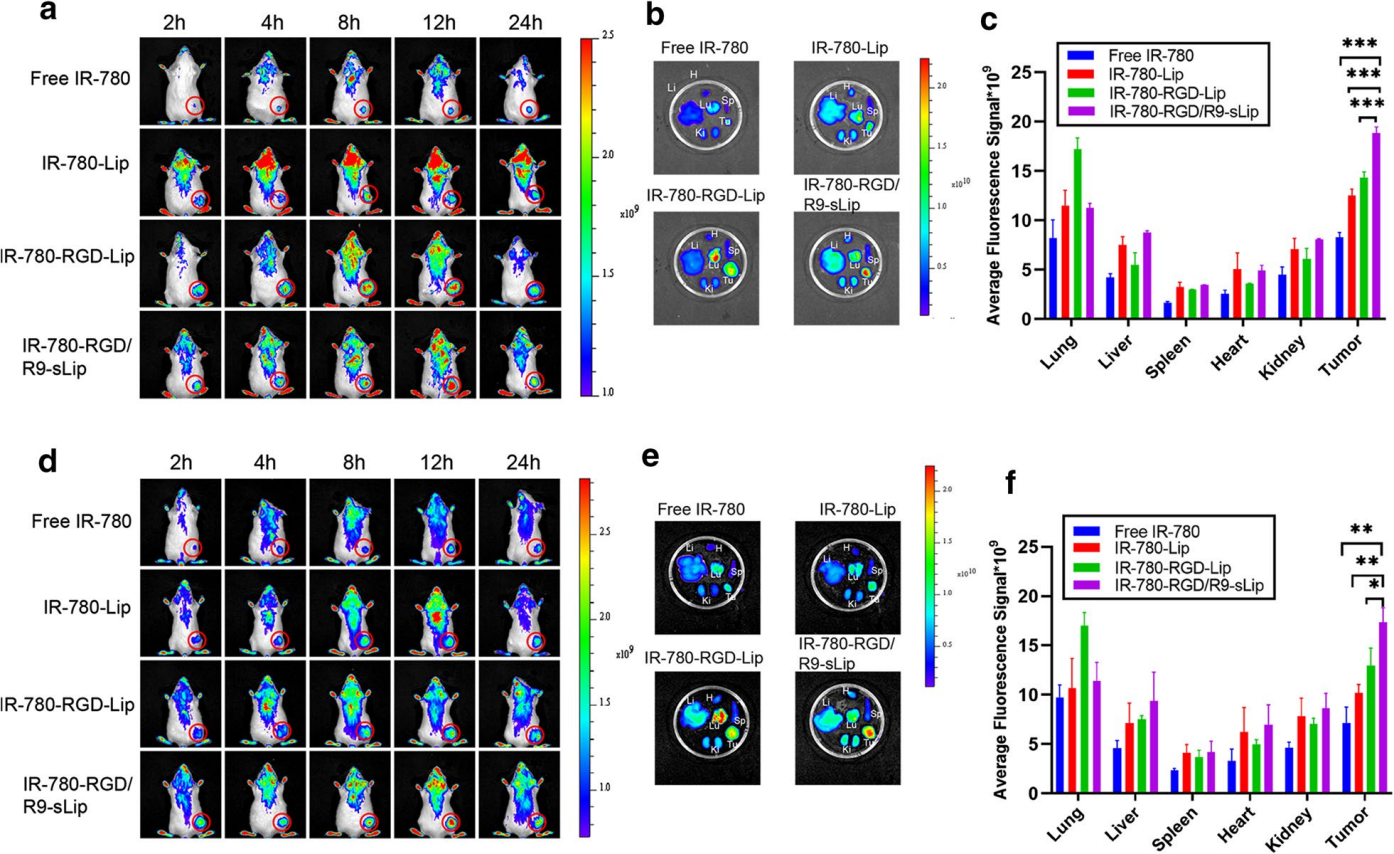
In vivo biodistribution of tumor-bearing mice. **a** Fluorescence signal distribution of CT-26 cell tumor-bearing model at 2, 4, 8, 12, and 24 h post-injection of Free IR-780, IR-780-Lip, IR-780-RGD-Lip and IR-780-RGD/R9-sLip. **b** Ex vivo fluorescence distribution of hearts (H), livers (Li), spleens (Sp), lungs (Lu), kidneys (Ki) and tumor (Tu). **c** Fluorescence in major organs and tumors was quantified using IVIS imaging software. **d** Fluorescence signal distribution of co-cultured cell tumor-bearing model injection of Free IR-780, IR-780-Lip, IR-780-RGD-Lip and IR-780-RGD/R9-sLip. **e** Ex vivo fluorescence distribution of hearts (H), livers (Li), spleens (Sp), lungs (Lu), kidneys (Ki) and tumor (Tu). **f** Fluorescence in major organs and tumors was quantified using IVIS imaging software. The red circles represent tumors. ^*^P < 0.05, ^**^P < 0.01, ^***^P < 0.001

In vivo biological distribution assay was performed to verify the distribution behaviors of liposomes in both CT-26 bearing mice model and CT-26/NIH 3T3 bearing mice model, respectively. In CT-26 bearing mice model (Fig. 5a–c), there were more tumor accumulation in all the liposomal formulations than free IR-780, which could be attributed to the EPR effect. Besides, the fluorescence intensity in free IR-780 group declined at 24 h, while strong signals sustained in the liposomal groups. Compared to the other liposomal formulations, enhanced fluorescence signals were observed at tumor sites in mice after treatment with IR-780-RGD-R9-sLip. It might be due to that after eliminating the barriers of CAFs by scFv, the liposomes could be more easily permeate and target into the deep tumor cells, herein increased tumor accumulation were observed. Furthermore, ex vivo imaging was carried out and quantified at 24 h post injection. The quantitative results revealed that IR-780-RGD/R9-sLip had higher fluorescence intensity than other formulations, due to the EPR effect and the dual targeting effect mediated by RGD and scFv. Similar results were also observed in co-cultured cells tumor-bearing mice as shown in Fig. 5d–f, in which IR-780-RGD/R9-sLip had enhanced tumor accumulation than the other groups.

In vivo distribution behaviors of liposomes were further monitored in orthotopic tumor model. Similar results were also observed in orthotopic tumor model as shown in Additional file 1: Fig. S7. Weak fluorescence was observed after administration with free IR-780, while all the liposomal groups showed increased fluorescent intensity, suggesting good long circulation of liposomes in colon cancer. Moreover, IR-780-RGD/R9-sLip exhibited stronger fluorescence than both IR-780-Lip and IR-780-RGD-Lip groups at all the timepoints, indicated that RGD/R9 modification would be beneficial for improving the accumulation of the cargoes into tumor site.

### In vivo antitumor effect in CT-26 tumor-bearing mice model

**Fig. 6:**
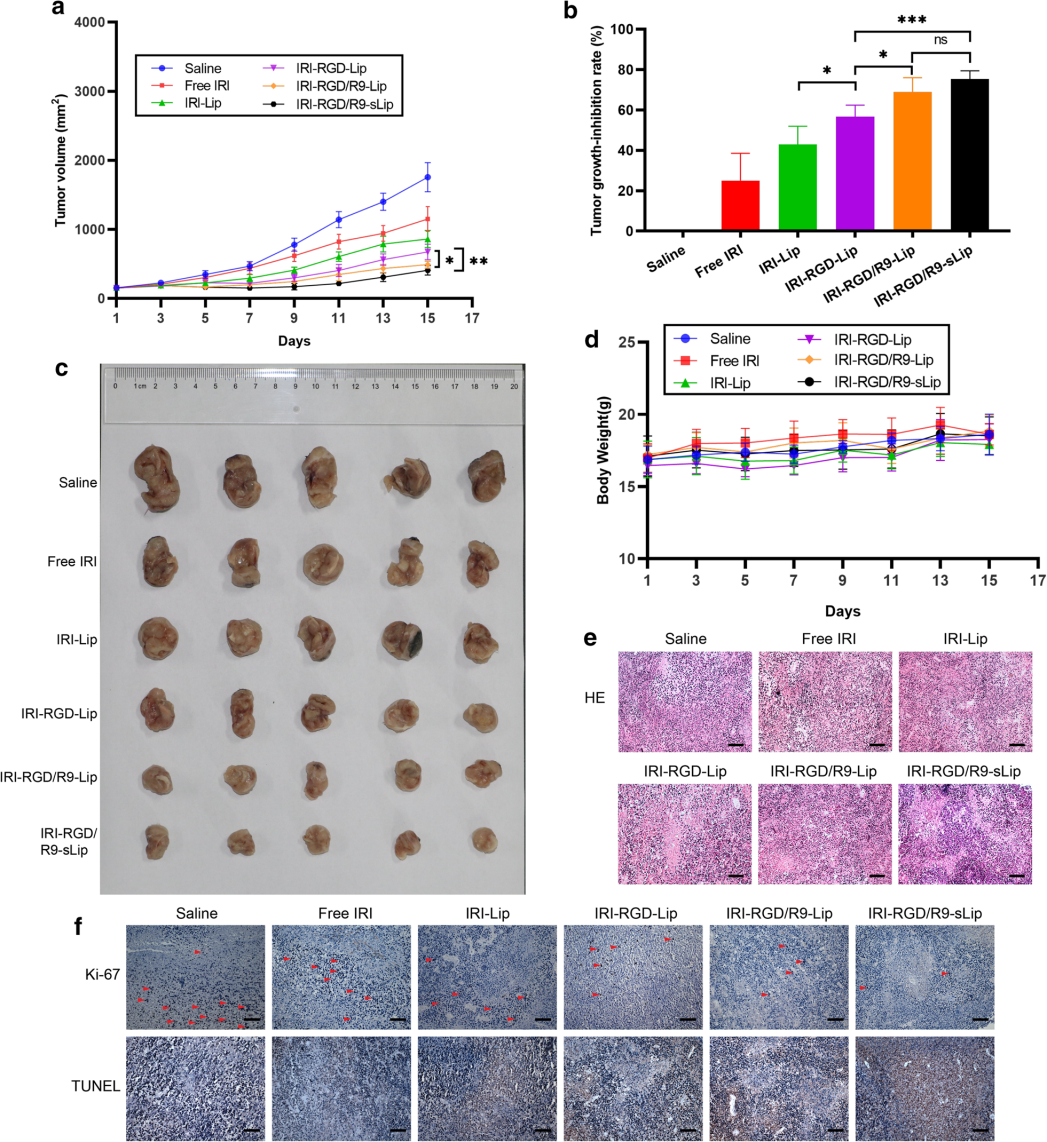
In vivo antitumor effect of subcutaneous inoculation of CT-26 cells in mouse model. **a** Tumor volume of mice in each treatment group changed. **b** Tumor growth-inhibition rate. **c** Excised tumor photographs. **d** Body weight change during treatment. **e** H&E staining and **f** Ki67 immunohistochemistry (arrow indicates positive proliferation), TUNEL staining (brown indicates apoptosis) of tumor tissue. Scale bar, 50 μm. Significance criteria: ^*^P < 0.05, ^**^P < 0.01, ^***^P < 0.001

The antitumor effect of different formulations was evaluated in CT-26 bearing mice model. Compared with the saline group, all the other preparation groups showed different degrees of tumor growth inhibition (Fig. 6a–c). In addition, after treatment with IRI-RGD-Lip, the anti-tumor effect was further enhanced with tumor growth inhibition rate 56.63%, which might be attributed to the increased cellular uptake via active targeting of RGD. Furthermore, the tumor inhibition rate of IRI-RGD/R9-Lip group (68.88%) was higher than that of the IRI-RGD-Lip group (*P* < 0.05), which may be attributed to the deep penetration ability of R9. However, compared with IRI-RGD/R9-Lip group, no significant difference (*P* > 0.05) existed in IRI-RGD/R9-sLip, indicating that in the tumor-bearing model of single tumor cells, the addition of scFv had limited therapeutic effect duo to the absence of CAFs.

**Fig. 7:**
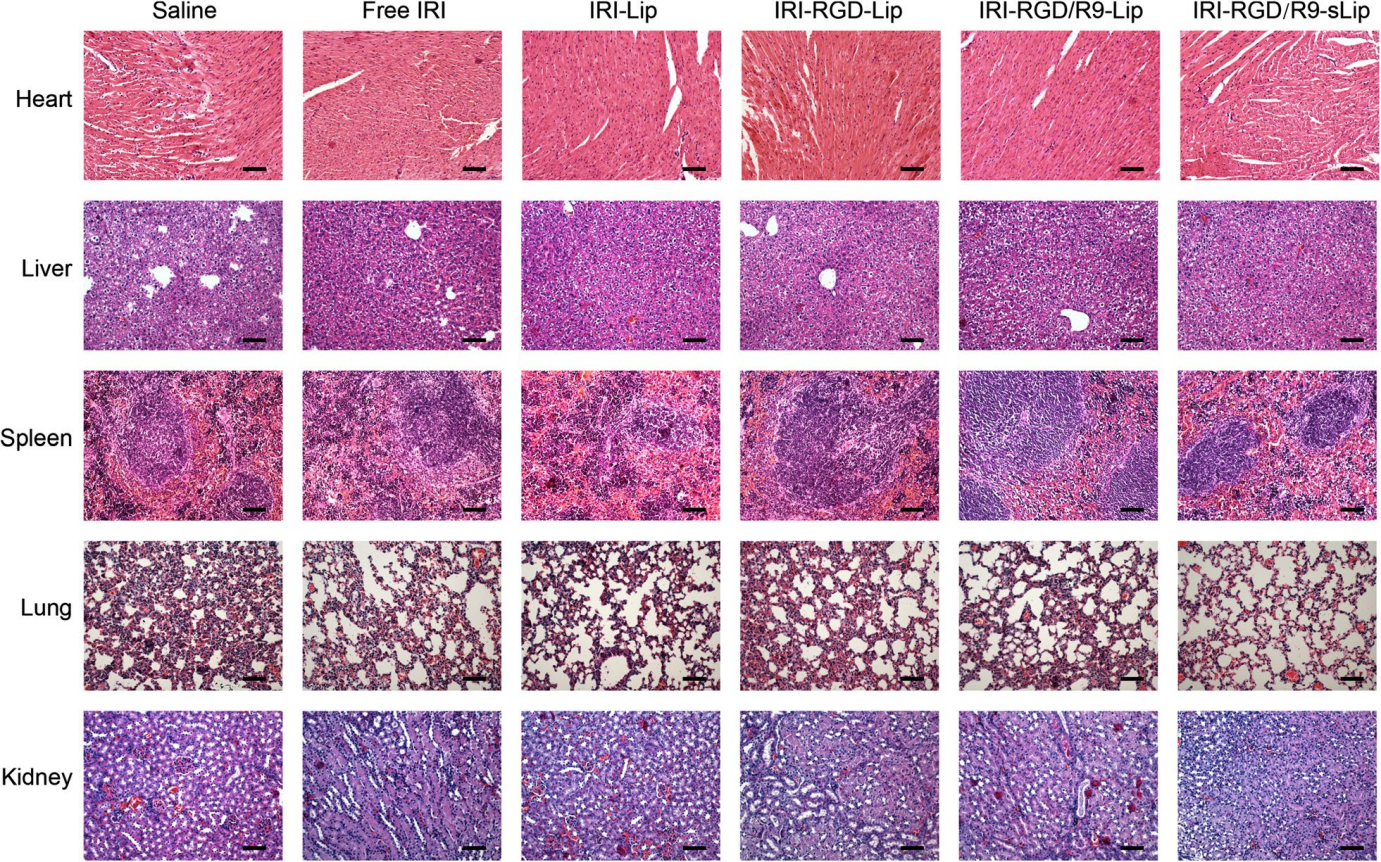
The organs of mice in different treatment groups were stained by H&E. Scale bar, 50 μm

There was no significant difference in body weights among all treatment groups as shown in Fig. 6d. Besides, no visualized tissue damage was observed in H&E section staining of major organs in mice, indicating that all preparations had no visible toxicities (Fig. 7). H&E staining of the tumor sections was also performed to further evaluate the status on tumor growth. More cells lysis after treatment by IRI-RGD/R9-sLip demonstrated that tumor proliferation was significantly inhibited. Consistent with H&E results, apoptosis was most obvious in the TUNEL experiment and the number of Ki67 positive cells was reduced in the IRI-RGD/R9-sLip treatment group (Fig. 6e, f).

### In vivo antitumor effect in CT-26/NIH 3T3 tumor-bearing mice model

**Fig. 8:**
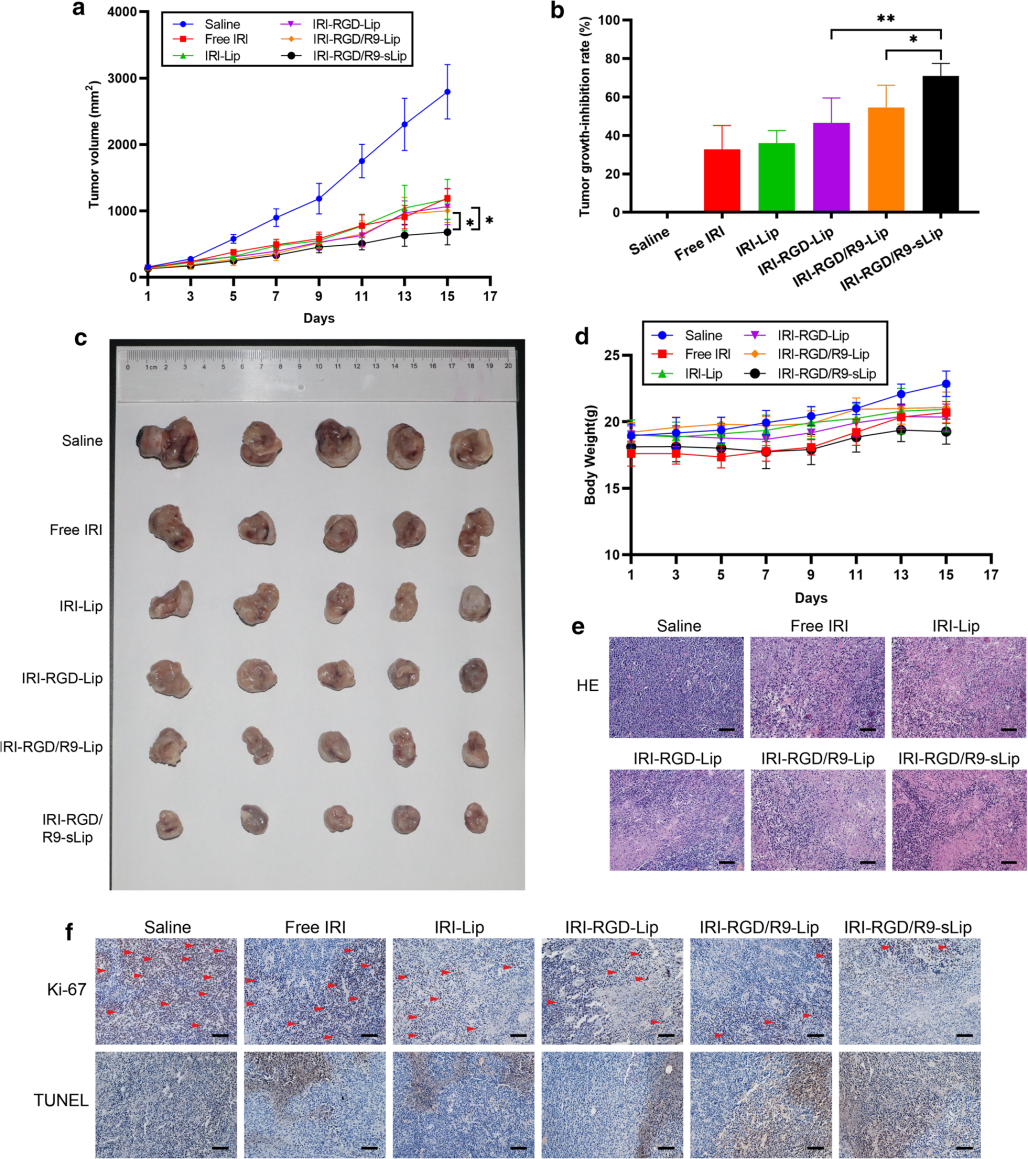
In vivo antitumor effect of subcutaneous inoculation of co-culture cells in mouse model. **a** Tumor volume of mice in each treatment group changed. **b** Tumor growth-inhibition rate. **c** Excised tumor photographs. **d** Body weight change during treatment. **e** H&E staining and **f** Ki67 immunohistochemistry (arrow indicates positive proliferation), TUNEL staining (brown indicates apoptosis) of tumor tissue. Scale bar, 50 μm. Significance criteria: ^*^P < 0.05, ^**^P < 0.01

In order to mimic the real CAFs-abundant tumor microenvironment, we further evaluated the antitumor effect in CT-26/NIH 3T3 co-cultured tumor-bearing mice model. As shown in Fig. 8a, at the end of administration, tumor volumes in the saline group reached ~ 3000 mm^3^, which was much higher than that in single CT-26 bearing mice model (about 2000 mm^3^). The faster tumor growth was attributed to the presence of a large number of stromal cells in the tumor microenvironment promoting the cell proliferation. IRI-RGD/R9-sLip exhibited superior anti-tumor advantages with the smallest tumor volumes compared to the other groups. Notably, the inhibition rate of IRI-RGD/R9-sLip group (70.97%) was higher than that of IRI-RGD/R9-Lip (54.38%) with significant difference (Fig. 8b), which was inconsistent with that in CT-26 bearing mice model. The results revealed that scFv could inhibit CAFs in the presence of a large number of CAFs, herein in favor of improving the antitumor effect. The tumor sizes also demonstrated that IRI-RGD/R9-sLip treatment could significantly inhibit the tumor growth (Fig. 8c). The above results suggested that in co-cultured tumor-bearing mice model, the combination of IRI and scFv could act on tumor cells and CAFs simultaneously to play a synergistic anti-tumor effect. Consequently, compared to the single tumor cells bearing mice model, co-culture cells would be a more reasonable model to reflect the real anti-tumor effect.

**Fig. 9:**
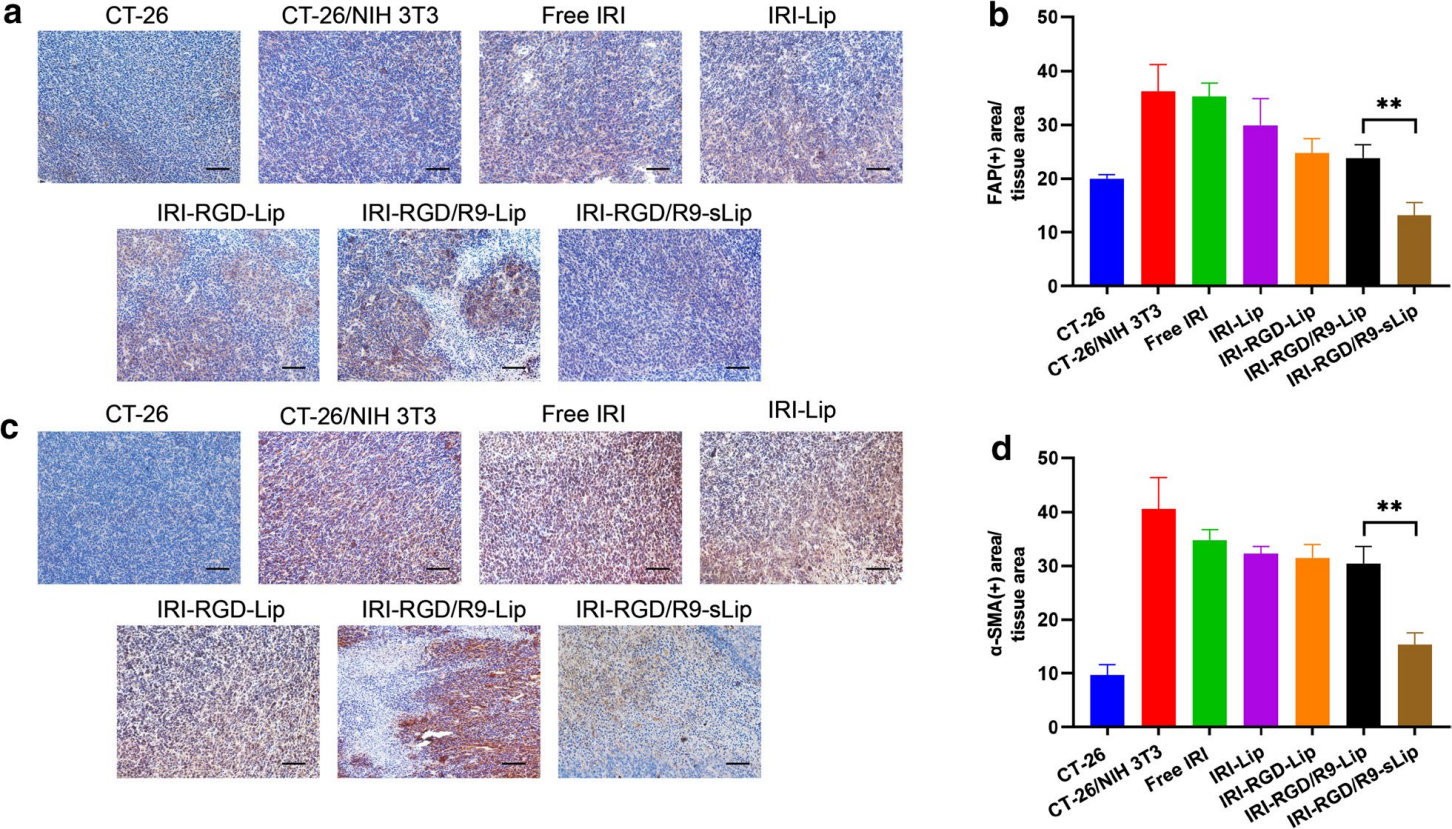
Immunohistochemical analysis of tumor. **a** Qualitative images and **b** quantitative analysis of FAP. **c** Qualitative images and **d** quantitative analysis of α-SMA in the tumor. Scale bar, 50 μm. Significance criteria: ^**^P < 0.01

Moreover, immunohistochemistry on H&E, TUNEL and Ki67 staining also showed the same results that the tumor sections in IRI-RGD/R9-sLip group had the most apoptotic and the least proliferative morphologies (Fig. 8e, f). The above results suggested that IRI combined with scFv has enhanced anti-tumor effect. Additionally, the expression of the biomarkers for CAFs including FAP and α-SMA was further evaluated by immunohistochemistry. As shown in Fig. 9a and c, compared to CT-26 tumor-bearing mice, both FAP and α-SMA expressions in CT-26/NIH 3T3 tumor-bearing mice were obviously increased, while significantly decreased after IRI-RGD/R9-sLip treatment. According to the quantitative analysis in Fig. 9b and d, IRI-RGD/R9-sLip had lower expression of FAP and α-SMA than the other groups, and there was significant difference compared to IRI-RGD/R9-Lip (*P* < 0.01). These results also verified that scFv could effectively kill CAFs and improve the anti-tumor effect of IRI-RGD/R9-sLip. The in vivo function of scFv was firstly investigated, although single scFv could inhibit the tumor growth, the effect was insufficient with growth inhibition rate less than 40% (Additional file 1: Fig. S8). In consideration of this, the combination of scFv and IRI would be more necessary, for which could co-act on both “seeds” and “soils” of the tumor, and thereby enhance the antitumor effect.

### In vivo antitumor effect in lung metastasis model and orthotopic tumor model

**Fig. 10:**
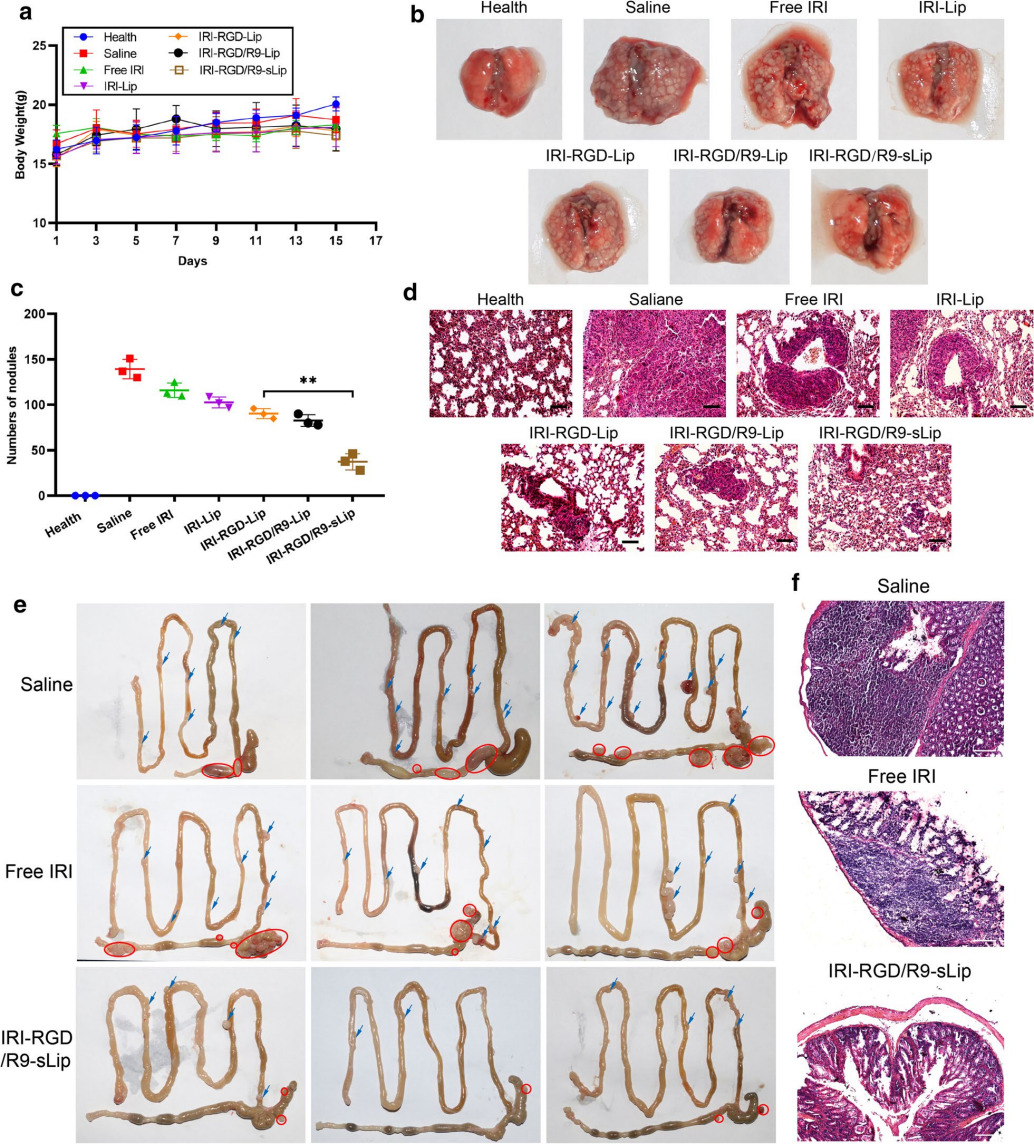
In vivo anti-metastatic effect. **a** Body weight change during treatment. **b** Representative photographs of excised lungs and **c** nodule numbers. **d** H&E staining of lung sections in each group. Scale bar, 50 μm. Significance criteria: ^*^P < 0.05, ^**^P < 0.01. **e** Excised tumor photographs of orthotopic tumor model. The red circle represents the primary tumor, while the blue arrow represents the site of metastatic tumors. **f** H&E staining of colon. Scale bar, 100 μm

The inhibition of tumor metastasis of different formulations was further studier by injecting CT-26 cells into tail vein of mice to establish lung metastasis model. As shown in the Fig. 10a, there was no significant weight loss during treatment, indicating that liposomes were provided with satisfactory biocompatibility and could be safe for drug delivery. Excised lung images of mice in each group were shown in Fig. 10b and the number of nodules was further quantified in Fig. 10c. Compared to free IRI, metastatic nodules in IRI-RGD-Lip group were reduced, which was due to the improved targeting of RGD modification. IRI-RGD/R9-sLip exhibited strongest anti-lung metastasis effect than the other groups (*P* < 0.01), which was attributed to the fact that IRI and scFv simultaneously killed tumor cells and CAFs. The histomorphology of lung was verified by H&E staining. As shown in Fig. 10d, all the treatment groups had different degrees of abnormal pulmonary cell aggregation, while IRI-RGD/R9-sLip treatment group had small tumor lesions based on the combined treatment, indicating superior anti-tumor effect of IRI-RGD/R9-sLip. These results demonstrated our design that the co-loaded liposomes could simultaneously kill both tumor cells and CAFs, thus improving the anti-tumor effect and inhibiting the metastasis.

Furthermore, the orthotopic colon cancer model was established in order to truly evaluate the anti-CRC effect. As shown in Fig. 10(e), depauperate colon and fast-growing primary tumors with large volumes could be observed in the saline group, meanwhile, there were also more metastatic tumors on the small intestine wall. Compared to saline group, both free IRI and liposomal groups exhibited efficient therapeutic effect with smaller tumor volumes. Notably, smaller volumes of primary tumor and less metastatic tumors could be observed after IRI-RGD/R9-sLip treatment than free IRI. Consequently, it could be deduced that IRI-RGD/R9-sLip could significantly inhibit tumor growth and scFv could inhibit CAFs in orthotopic tumor model. Additionally, the colon tissues were further sectioned for H&E staining (Fig. 10f). There was a larger area of tumor invasion in the saline group, while the tumor invasion site decreased after IRI treatment. In contrast, no obvious invasion was observed in IRI-RGD/R9-sLip group. These results suggested that IRI-RGD/R9-sLip could significantly improve the therapeutic effect.

## Conclusions

In this study, the novel co-loaded multifunctional liposomes were developed that integrated multiple advantages, including IRI and scFv co-loading, scFv targeting and killing CAFs, RGD mediated active targeting, R9 improving the deep penetration of liposomes and lysosomal escape function, which could act on the tumor cells and destroy the "soil" of tumor cell survival simultaneously, and thus effectively reduce the metastasis and recurrence of malignant tumor. IRI-RGD/R9-sLip showed enhanced cytotoxicity in different cell models in vitro, effectively increased the accumulation in tumor sites in vivo and also exhibited deep permeation ability both in vitro and in vivo. Notably, IRI-RGD/R9-sLip not only exhibited superior in vivo anti-tumor effect in both CAFs-free and CAFs-abundant bearing mice models, but also presented excellent anti-metastasis efficiency in lung metastasis model. In conclusion, the combinational strategy on killing both tumor cells and CAFs provides a new approach for cancer therapy, and the prepared co-loaded liposomes hold promising clinical application prospects.

## Supplementary Information

**Additional file 1: Table S1**. Characterizations of DOX-loaded and IR780-Liposomes. **Fig. S1**. 1H-NMR spectra characterization of (a) DSPE-PEG-RGD and (b) DSPE-PEG-R9. **Fig. S2**. Cell viability of (a) CT-26 cells or (b) co-cultured cells (ratio of CT-26 to NIH 3T3 cells was 1:2) after incubation with blank liposome, RGD-Lip and RGD/R9-Lip at different concentrations for 48 h. **Fig. S3**. Cell viability of activated NIH 3T3 cells after incubation with RGD/R9-Lip and RGD/R9-sLip for 48 h. **Fig. S4**. Cell viability of activated NIH 3T3 cells after incubation with IRI for 48 h. **Fig. S5**. The colocalization ratio of lysosome and liposome was quantified using Image J. *P <0.05, **P < 0.01. **Fig. S6**. The release profile of scFv after incubated with activated NIH3T3 cells. **Fig. S7**. In vivo biodistribution of orthotopic tumor model. (a) Fluorescence signal distribution of orthotopic tumor model at 2, 4, 8, 12, and 24 h post-injection of Free IR-780, IR-780-Lip, IR-780-RGD-Lip and IR-780-RGD/R9-sLip. (b) Ex vivo fluorescence distribution of hearts (H), livers (Li), spleens (Sp), lungs (Lu), kidneys (Ki) and colon tumor (CT). **Fig. S8**. In vivo antitumor effect of subcutaneous inoculation of co-culture cells in mouse model. (a) Tumor volume of mice in each treatment group changed. (b) Tumor growth-inhibition rate. (c) Body weight change during treatment. (d) Excised tumor photographs. (e) Immunohistochemical analysis of tumor tissue. Scale bar, 50 μm.

**Additional file 2**. A representive video of 3D imaging in Fig. 4(d).

## Data Availability

All data generated or analyzed during this study are included in this published article.
