# Supplementary material for: Irinotecan/scFv co-loaded liposomes coaction on tumor cells and CAFs for enhanced colorectal cancer therapy
Source: J Nanobiotechnology. 2021 Dec 14;19:421. doi: 10.1186/s12951-021-01172-0 (PMC8670172; doi:10.1186/s12951-021-01172-0)
Supplement: Supplementary file 1 — Additional file 1: Table S1. Characterizations of DOX-loaded and IR780-Liposomes. Fig. S1. 1H-NMR spectra characterization of (a) DSPE-PEG-RGD and (b) DSPE-PEG-R9. Fig. S2. Cell viability of (a) CT-26 cells or (b) co-cultured cells (ratio of CT-26 to NIH 3T3 cells was 1:2) after incubation with blank liposome, RGD-Lip and RGD/R9-Lip at different concentrations for 48 h. Fig. S3. Cell viability of activated NIH 3T3 cells after incubation with RGD/R9-Lip and RGD/R9-sLip for 48 h. Fig. S4. Cell viability of activated NIH 3T3 cells after incubation with IRI for 48 h. Fig. S5. The colocalization ratio of lysosome and liposome was quantified using Image J. *P <0.05, **P < 0.01. Fig. S6. The release profile of scFv after incubated with activated NIH3T3 cells. Fig. S7. In vivo biodistribution of orthotopic tumor model. (a) Fluorescence signal distribution of orthotopic tumor model at 2, 4, 8, 12, and 24 h post-injection of Free IR-780, IR-780-Lip, IR-780-RGD-Lip and IR-780-RGD/R9-sLip. (b) Ex vivo fluorescence distribution of hearts (H), livers (Li), spleens (Sp), lungs (Lu), kidneys (Ki) and colon tumor (CT). Fig. S8. In vivo antitumor effect of subcutaneous inoculation of co-culture cells in mouse model. (a) Tumor volume of mice in each treatment group changed. (b) Tumor growth-inhibition rate. (c) Body weight change during treatment. (d) Excised tumor photographs. (e) Immunohistochemical analysis of tumor tissue. Scale bar, 50 μm. [file 12951_2021_1172_MOESM1_ESM.docx]

**Irinotecan/scFv co-loaded liposomes coaction on tumor cells and CAFs for enhanced colorectal cancer therapy**

Zhaohuan Li^1^^#^, Chunxi Liu^2#^, Chenglei Li^1^, Fangqing Wang^3^, Jianhao Liu^1^, Zengjuan Zheng^1^, Jingliang Wu^3*^, Bo Zhang^1*^

^1^ School of Pharmacy, Weifang Medical University Weifang, Shandong, 261053, P.R. China

^2^ Department of Pharmacy, Qilu Hospital, Cheeloo College of Medicine, Shandong University, Ji'nan, Shandong, 250012, P.R. China

^3^ School of Bioscience and Technology, Weifang Medical University, Weifang, Shandong, 261053, P.R. China

^#^Zhaohuan Li and Chunxi Liu contributed equally to this work

^*^Corresponding authors: jlwu2008@163.com, zhangbo@wfmc.edu.cn.

Table S1 Characterizations of DOX-loaded and IR780-Liposomes

| Formulations | DLS(nm) | PDI | Formulations | DLS(nm) | PDI |
| --- | --- | --- | --- | --- | --- |
| DOX-Lip | 133.9±3.1 | 0.18±0.15 | IR780-Lip | 135.6±2.3 | 0.20±0.02 |
| DOX-RGD-Lip | 133.2±2.5 | 0.17±0.04 | IR780-RGD-Lip | 135.0±0.2 | 0.20±0.04 |
| DOX-RGD/R9-Lip | 132.4±3.2 | 0.19±0.02 | IR780-RGD/R9-sLip | 132.0±3.9 | 0.18±0.04 |


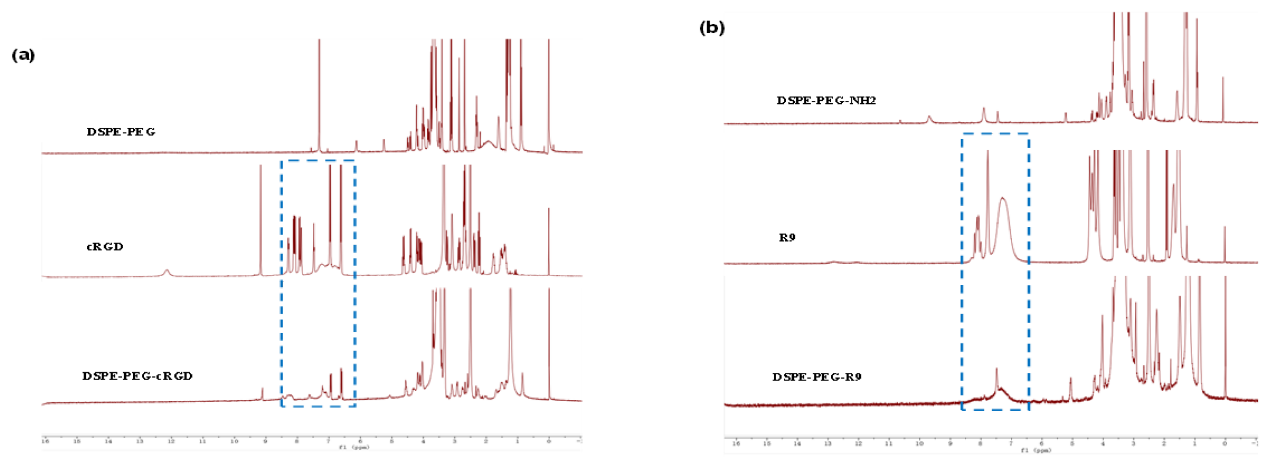


Fig. S1. ^1^H-NMR spectra characterization of (a) DSPE-PEG-RGD and (b) DSPE-PEG-R9.


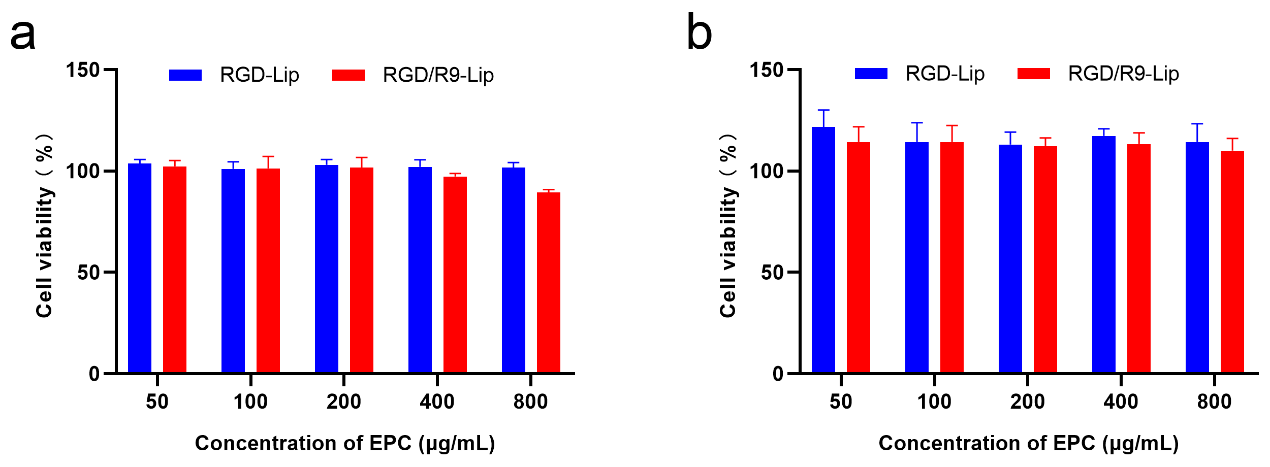


Fig. S2. Cell viability of (a) CT-26 cells or (b) co-cultured cells (ratio of CT-26 to NIH 3T3 cells was 1:2) after incubation with blank liposome, RGD-Lip and RGD/R9-Lip at different concentrations for 48 h.


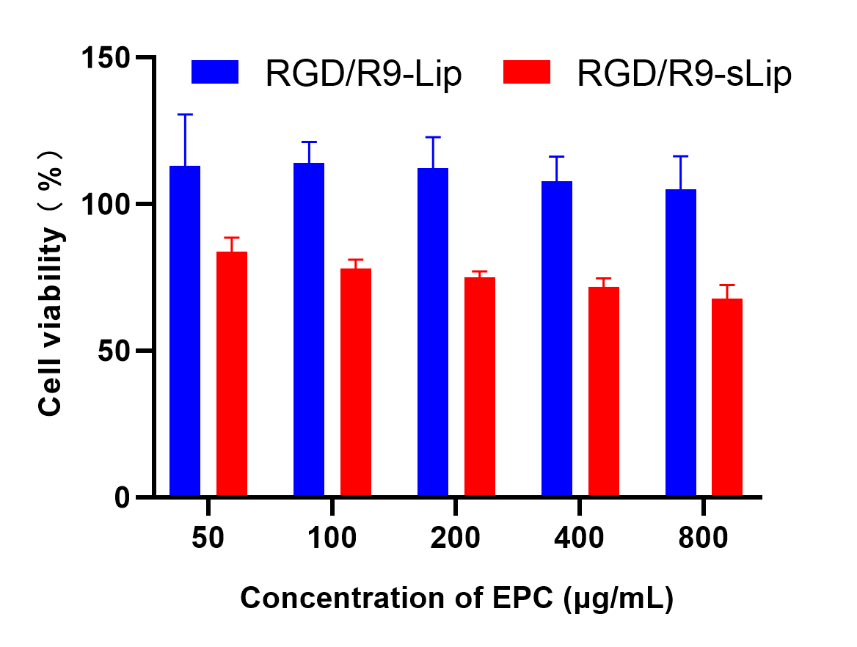


Fig. S3. Cell viability of activated NIH 3T3 cells after incubation with RGD/R9-Lip and RGD/R9-sLip for 48 h.


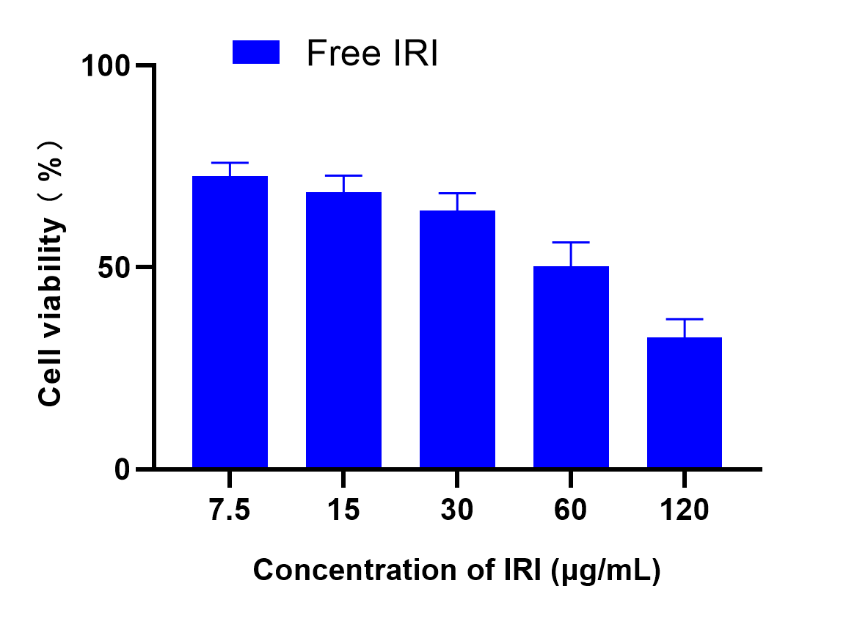


Fig. S4. Cell viability of activated NIH 3T3 cells after incubation with IRI for 48 h.


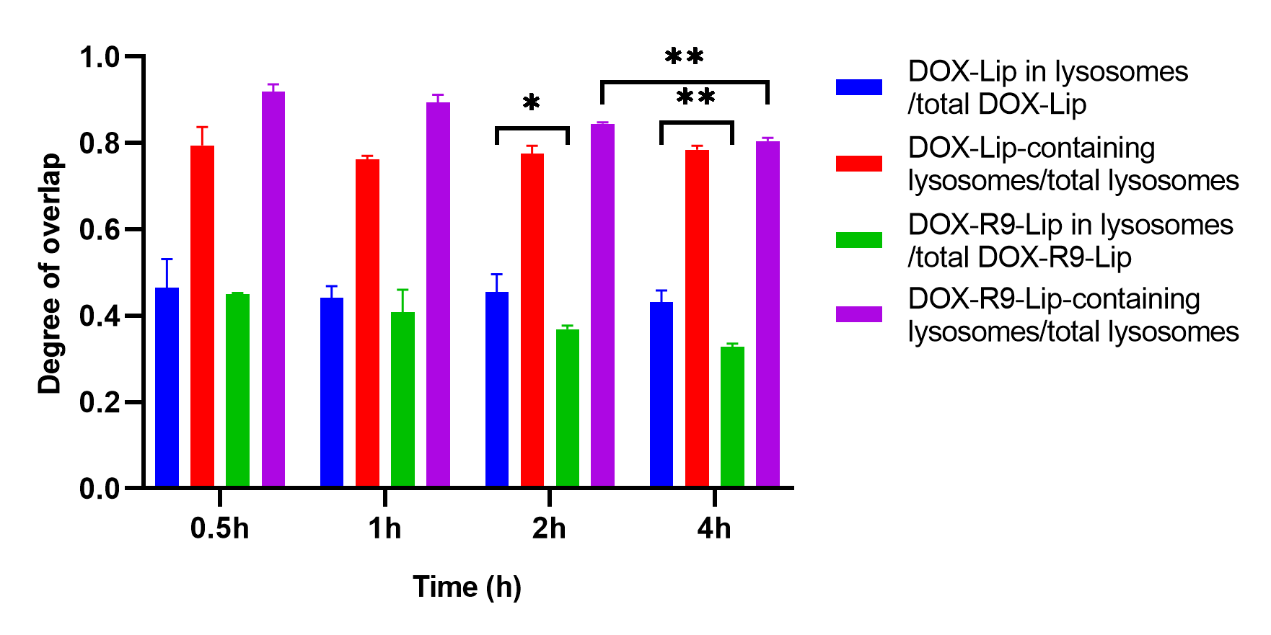


Fig S5. The colocalization ratio of lysosome and liposome was quantified using Image J. **P <0.05*, ***P < 0.01*.


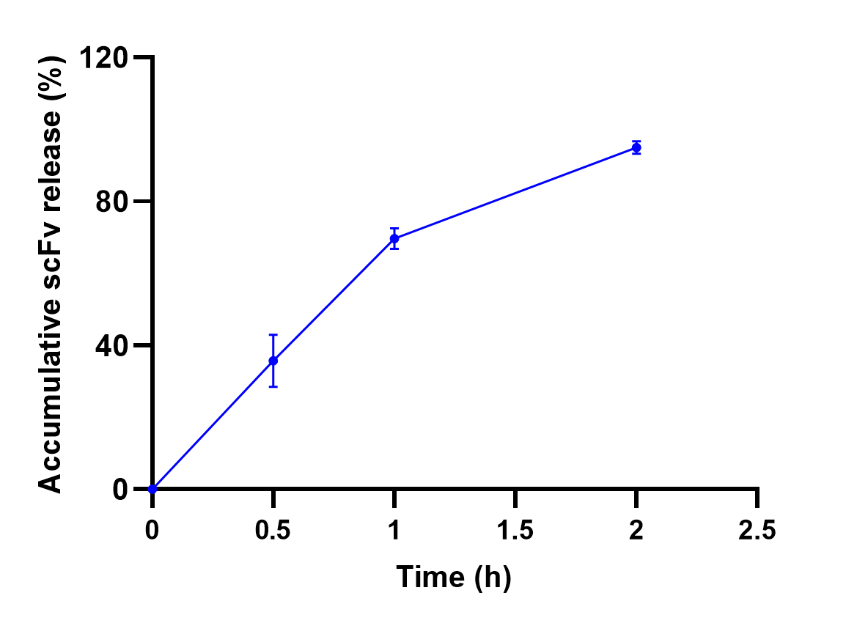


Fig. S6. The release profile of scFv after incubated with activated NIH3T3 cells.


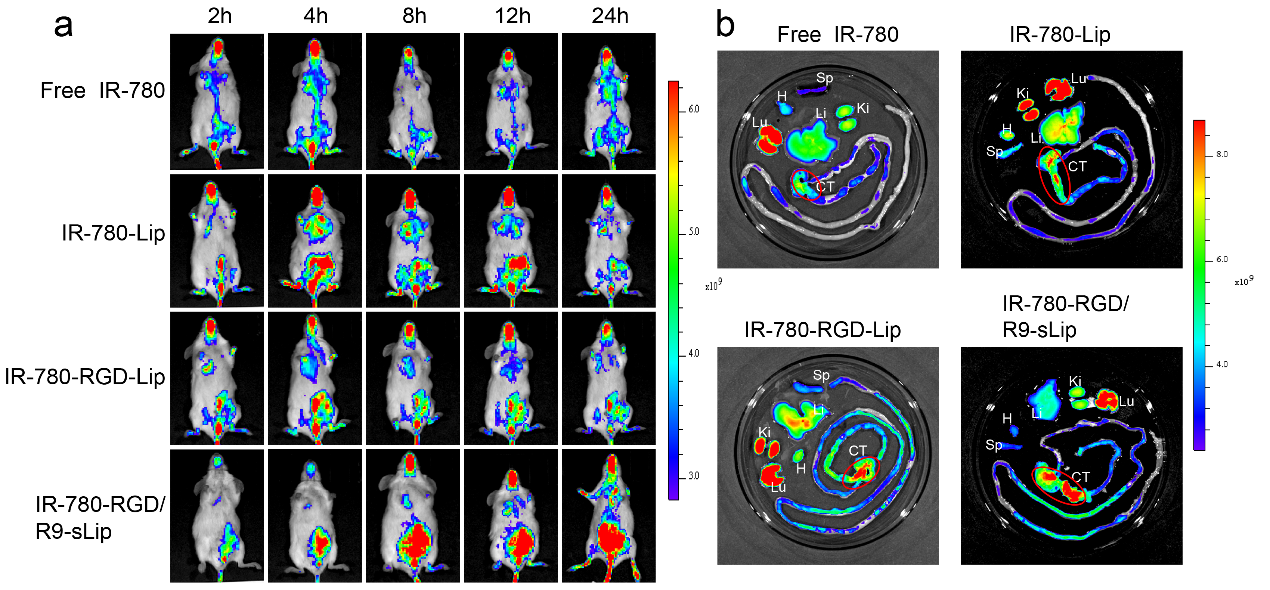


Fig S7. *In vivo* biodistribution of orthotopic tumor model. (**a**) Fluorescence signal distribution of orthotopic tumor model at 2, 4, 8, 12, and 24 h post-injection of Free IR-780, IR-780-Lip, IR-780-RGD-Lip and IR-780-RGD/R9-sLip. (**b**) *Ex vivo* fluorescence distribution of hearts (H), livers (Li), spleens (Sp), lungs (Lu), kidneys (Ki) and colon tumor (CT).


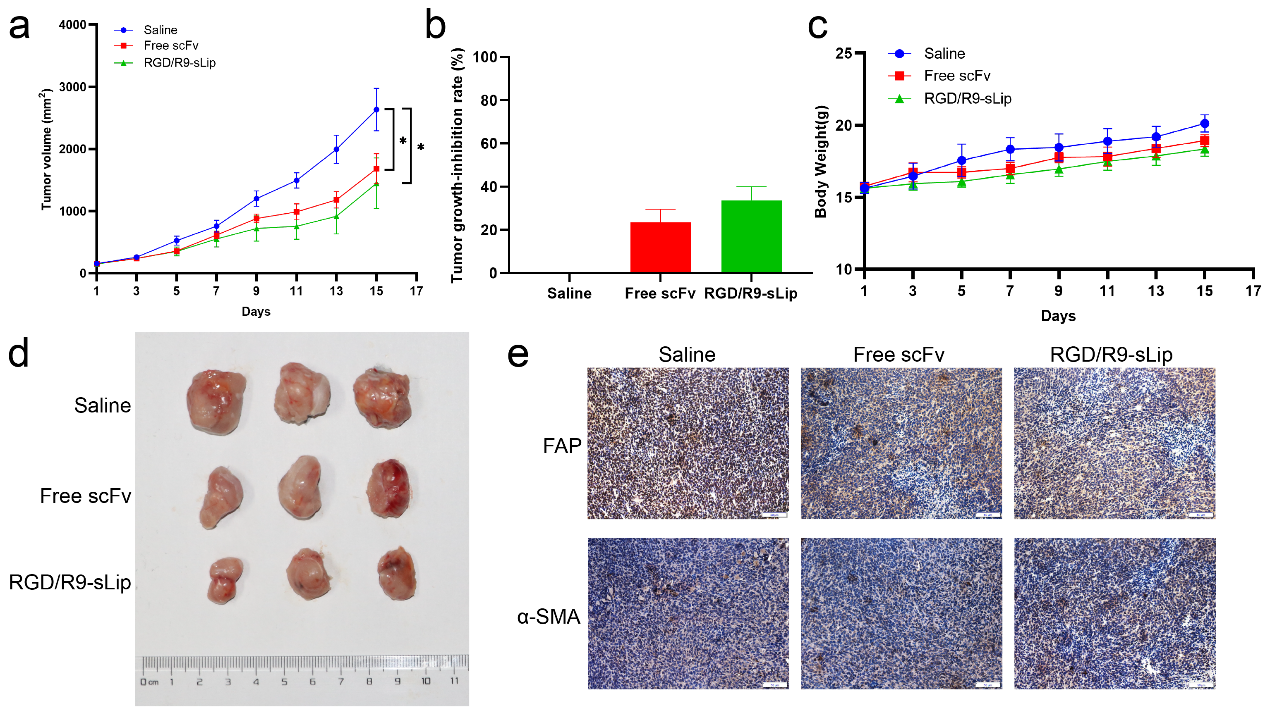


Fig S8. *In vivo* antitumor effect of subcutaneous inoculation of co-culture cells in mouse model. (a) Tumor volume of mice in each treatment group changed. (b) Tumor growth-inhibition rate. (c) Body weight change during treatment. (d) Excised tumor photographs. (e) Immunohistochemical analysis of tumor tissue. Scale bar, 50 μm.
